# Evaluating the In Vitro Potential of Natural Extracts to Protect Lipids from Oxidative Damage

**DOI:** 10.3390/antiox9030231

**Published:** 2020-03-11

**Authors:** Rafael Félix, Patrícia Valentão, Paula B. Andrade, Carina Félix, Sara C. Novais, Marco F. L. Lemos

**Affiliations:** 1 MARE—Marine and Environmental Sciences Centre, ESTM, Instituto Politécnico de Leiria, 2520-641 Peniche, Portugal; carina.r.felix@ipleiria.pt (C.F.); sara.novais@ipleiria.pt (S.C.N.); marco.lemos@ipleiria.pt (M.F.L.L.); 2 REQUIMTE/LAQV, Laboratório de Farmacognosia, Faculdade de Farmácia, Universidade do Porto, 4050-313 Porto, Portugal; valentao@ff.up.pt (P.V.); pandrade@ff.up.pt (P.B.A.)

**Keywords:** lipid peroxidation, LPIP, natural products, bioactive compounds, in vitro

## Abstract

Lipid peroxidation is a chemical reaction known to have negative impacts on living organisms’ health and on consumer products’ quality and safety. Therefore, it has been the subject of extensive scientific research concerning the possibilities to reduce it, both in vivo and in nonliving organic matrices. It can be started by a variety of oxidants, by both ROS-dependent and -independent pathways, all of them reviewed in this document. Another feature of this reaction is the capacity of lipid peroxyl radicals to react with the non-oxidized lipids, propagating the reaction even in the absence of an external trigger. Due to these specificities of lipid peroxidation, regular antioxidant strategies—although being helpful in controlling oxidative triggers—are not tailored to tackle this challenge. Thus, more suited antioxidant compounds or technologies are required and sought after by researchers, either in the fields of medicine and physiology, or in product development and biotechnology. Despite the existence of several laboratory procedures associated with the study of lipid peroxidation, a methodology to perform bioprospecting of natural products to prevent lipid peroxidation (a Lipid Peroxidation Inhibitory Potential assay, LPIP) is not yet well established. In this review, a critical look into the possibility of testing the capacity of natural products to inhibit lipid peroxidation is presented. In vitro systems used to peroxidize a lipid sample are also reviewed on the basis of lipid substrate origin, and, for each of them, procedural insights, oxidation initiation strategies, and lipid peroxidation extent monitoring are discussed.

## 1. Introduction

Oxidation is a well-known factor affecting the integrity of biological systems, influencing the physiological state of living organisms and the chemical composition of organic matrices. Oxidative damage can be induced by a variety of physicochemical processes, such as exposure to heat, light, or oxidizing chemical agents [1,2]. One of the most relevant classes of oxidizing chemical agents causing oxidation of biomolecules are Reactive Oxygen Species (ROS), which are ubiquitous in biological tissues due to aerobic metabolism or to direct exposure to the atmosphere [3,4]. While affecting most types of molecules, oxidation occurrence in lipids (lipid peroxidation, LPO) is a problem for both human health and the industries of food, feed, and cosmetic products.

Lipid peroxidation in cells results in the degradation of the lipid bilayer composing cell membranes [5,6]. Besides, LPO end-products can further promote mutagenesis or protein oxidation, disturbing cellular homeostasis, and their implications in human health have been extensively reviewed [7,8,9,10,11,12,13]. In nonliving biological matrices (organic products, such as food or feeds), LPO is known to cause nutritional-value loss, the development of unpleasant sensorial characters (such as the rancid off-flavors and darkening of food products), and the occurrence of by-products toxic to the consumer [14,15,16]. Therefore, inhibiting LPO is of utmost importance for industries interested in both health-promoting applications and shelf-life-extending technologies. However, despite the paramount need for preservatives, specifically for food products, the use of synthetic antioxidants as additives in product formulas has been increasingly discouraged by policy makers and consumers, given their proven/potential negative effects on human health and the environment [17]. Natural substituents are therefore sought by industrial manufacturers of LPO-prone products. Additionally, antioxidant-containing food products are increasingly demanded by the fast-growing market of healthy foods.

Screening for these natural-sourced antioxidants has become a common practice in bioprospecting/biotechnology. Many biomass sources are widely recognized as containing natural antioxidants (mostly of phenolic nature) and are tentatively explored as additives (either as whole-ingredients or in the form of extracts/isolated molecules) in product formulations [18,19,20,21,22,23,24]. However, not every natural extract, fraction, or molecule (from here on referred to as natural product, NP) with antioxidant capacity will exhibit the same activity in protecting lipid substrates from peroxidation, as it depends on several factors such, as redox potential and lipophilicity of the compounds [25]. Effective antioxidants can protect against lipid peroxidation through different modes of actions: They can act indirectly, by neutralizing one of the initiators of the lipid oxidative damage (e.g., by absorbing photons, neutralizing ROS, or chelating metal ions), or directly, by neutralizing lipid radicals (the first product of lipid oxidative damage), thereby stopping the propagation reactions, typical of lipid peroxidation. Thus, complex natural extracts will exhibit a combined Lipid Peroxidation Inhibitory Potential (LPIP), depending on their composition on antioxidants capable of neutralizing oxidative stress and stopping the chain reaction of lipid radical propagation. 

The indirect antioxidant potential of biological extracts can be screened by a variety of in vitro methodologies, most of which are dedicated to only one of the triggers presented above. Long-known assays that are very common in the literature of bioactive compounds (particularly antioxidants), such as the reduction of diphenylpicrylhydrazyl (DPPH), the ferric-reducing antioxidant potential (FRAP), or the oxygen radical absorbing capacity (ORAC), are some examples of the wide variety of antioxidant methods that will indirectly reflect an extract’s LPIP. This thematic of antioxidants has been extensively reviewed elsewhere [26,27,28,29]. Because each of these assays are specific for one of the triggers, none of them reflects the combined capacity of NPs to mitigate LPO altogether, whether by exerting effect in more than one trigger or by acting on a different phase of the LPO reactions (e.g., neutralizing the lipid peroxyl radical, thereby stopping the chain reaction of LPO). However, when analyzing LPIP by using an in vitro model of LPO, the combined effects of indirect and direct modes of action of antioxidants can be determined, broadening the spectra of possible successful hits in NPs’ biodiscovery. Therefore, LPIP in vitro assays are the focus of this review, and the scientific literature on this subject is further addressed and detailed in Section 3.

## 2. Lipid Peroxidation

Lipid peroxidation is a type of oxidative degradation of biomolecules, where a peroxide is formed from a lipid substrate. Peroxides are compounds containing a structural formula of the type R-O-O-R’, where O are oxygen atoms in the oxidative state −1, a less common and less stable form of oxygen. Lipids are especially prone to peroxidation, mainly the polyunsaturated fatty acids (PUFAs), whether in the form of triacylglycerides (TAGs) or of free fatty acids (FFAs), but also polar lipids containing these PUFAs (mostly glycolipids, phospholipids, and sphingolipids), and cholesterol, due to the presence of methylene groups adjacent to double bonds [30]. In this section, the reaction mechanism of LPO is briefly discussed, with the goal of introducing two main concepts of extreme relevance to this review of methods to determine LPIP in vitro: initiators and end-products. These two concepts support the discussion of two important steps in any method that attempts to measure LPIP: experimental induction of the oxidation in the model substrate and the experimental detection of the oxidation extent, respectively.

### 2.1. On the Mechanism of Lipid Peroxidation

**Figure 1:**
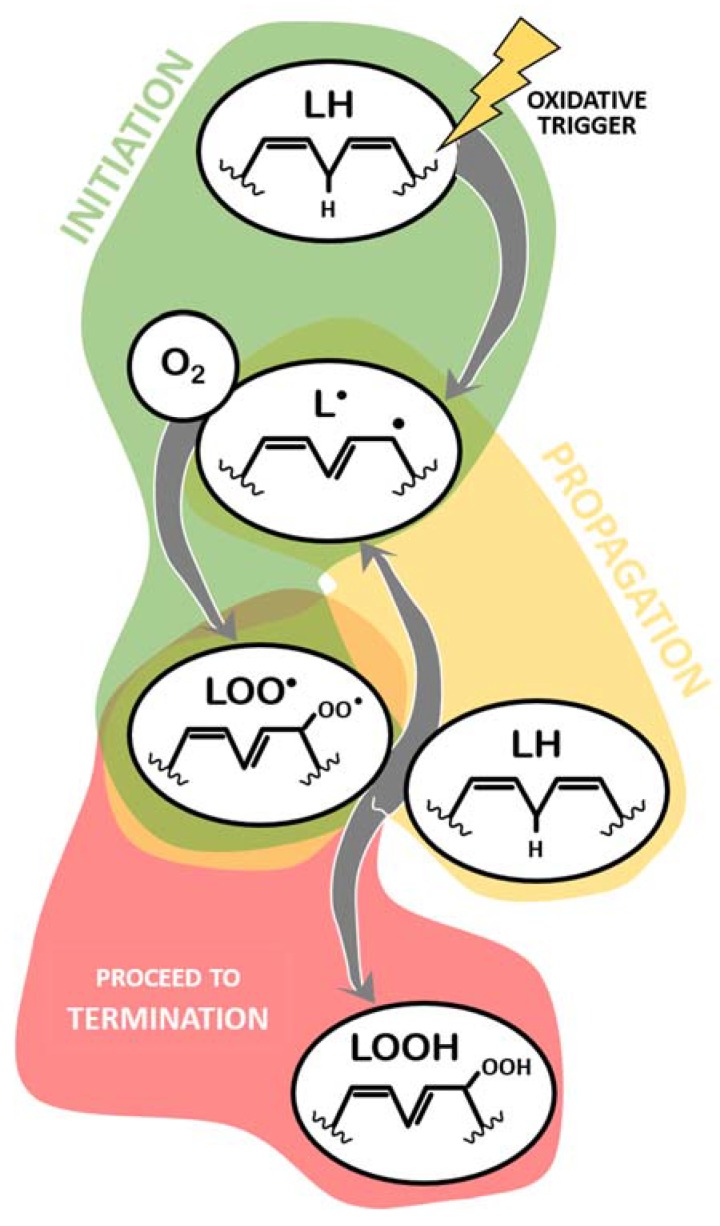
Main molecules involved in the initiation and propagation of lipid peroxidation. LH: lipid molecule; LOO^●^: lipid peroxyl radical; L^●^: lipid radical; LOOH: lipid hydroperoxide.

**Figure 2:**
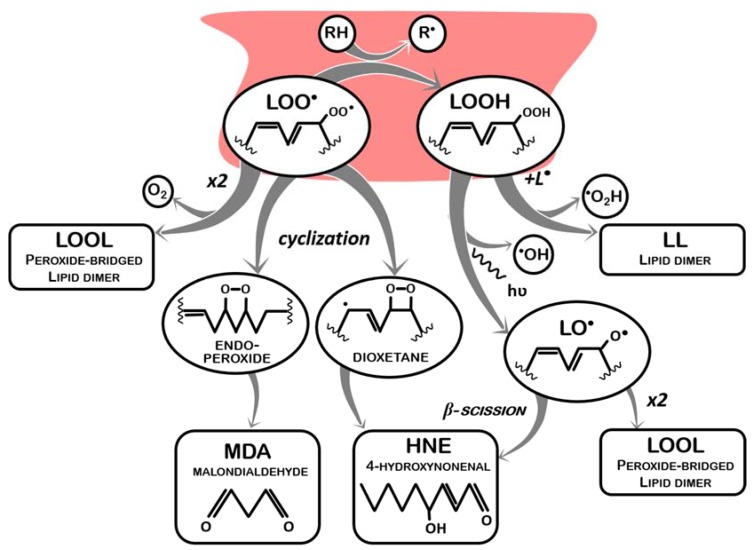
Main molecules involved in the termination of lipid peroxidation. RH: proton-donor; R^●^: radical.

The mechanism of LPO has been extensively characterized [30,31,32,33,34,35,36,37], and a summary of different aspects relevant to this review is provided below. Every LPO reaction can be generically described as a three-step process (Figure 1 and Figure 2): initiation, propagation, and termination. Initiation of LPO refers to the moment when a given oxidative trigger contacts with the lipid molecule (LH, Figure 1) and causes the loss of a hydrogen atom from a methylene group, thus creating a carbon radical lipid (L^●^) [36]. Here, this part of the process is generically described as LH -> L^●^ and is analyzed below, in Section 2.2.

The created lipid radical rapidly reacts with available molecular oxygen (O_2_) to form a lipid peroxyl radical (LOO^●^, Figure 1), acquiring both atoms of oxygen at the carbon radical (L^●^ + O_2_ -> LOO^●^). Before proceeding to an explanation of the remaining process, it is pertinent to note that, upon formation, the peroxyl radical suffers a rearrangement and becomes a conjugated diene. This is only not true if the lipid molecule being oxidized is a saturated fatty acid (extremely unlikely) or a monounsaturated fatty acid (MUFA) (more likely than saturated, but less likely than others containing methylene groups in between double bonds, namely PUFAs) [38]. This information is relevant when discussing the methods to detect ongoing lipid peroxidation.

This lipid peroxyl radical has now one of two main fates: propagation, in which it reacts with another lipid molecule containing a methylene group, abstracting a proton from this second molecule (LOO^●^ + LH -> LOOH + L^●^), thus becoming a lipid hydroperoxide (LOOH) and generating a new lipid radical (and the cycle restarts, which can happen indefinitely) (Figure 1); or termination, in which lipid peroxyl radicals and lipid hydroperoxides are neutralized. Termination of lipid peroxidation begins by neutralizing LOO^●^. In this sense, during the propagation phase, despite the generation of a new L^●^ that will fuel more LPO, one of the reactive lipids is “terminated”. This process involves other reactions of LOO^●^ molecules not resulting in the oxidation of another lipid, as schematized in Figure 2 and listed below:

(a) It receives a proton from a proton-donor (RH) other than another lipid (LOO^●^ + RH -> LOOH + R^●^), such as an endogenous antioxidant (vitamin A or vitamin E), thus generating a lipid hydroperoxide (LOOH) and another radical (R^●^), which may be less reactive and have its own pathway of degradation (in case of endogenous antioxidant systems, enzymatically or not, these molecules can be restored without causing further damage);
(b) It reacts with another lipid peroxyl radical to form a peroxide bridged dimmer (LOO^●^ + LOO^●^ -> LOOL + O_2_);
(c) It undergoes peroxycyclization, which mediates the decay of LOO^●^ into malondialdehyde (MDA) and 4-hydroxynonenal (HNE), the best-known LPO by-products.

Either from propagation or from termination by an antioxidant system, LOOH generated also represents an issue, as it is still an unstable molecule that readily reacts as follows:

(a) One LOOH reacts with one L^●^ to form a lipid dimer (LOOH + L^●^ -> LL) and hydroperoxyl radicals (HO_2_^●^) are released;
(b) One LOOH decays into a lipid alkoxyl radical (LO^●^), and hydroxyl radicals (^●^OH) are released; lipid alkoxyl radicals (LO^●^) can undergo β-scission, originating smaller radicals (such as pentane^●^ and ethane^●^) and HNE.

Noticeably, LOO^●^ and LOOH (created by an oxidative trigger) not only behave like oxidants, reacting with intact lipids and with each other, but also regenerate free radicals when they finally become stabilized (which further amplifies oxidative damages). Furthermore, if dimmers are not formed, and stabilization occurs instead via fragmentation of the lipid chain, several small toxic metabolites (aldehydes, ketones, and carboxylic acids, among others) are formed. Two of the most important ones are MDA and HNE, which present negative impacts in both organism fitness and product quality and safety, by causing additional damages to biomolecules (proteins and DNA) and the off-flavors associated to edible fats rancidification [14,15,36,37,39].

### 2.2. On the Initiators of Lipid Peroxidation

**Figure 3:**
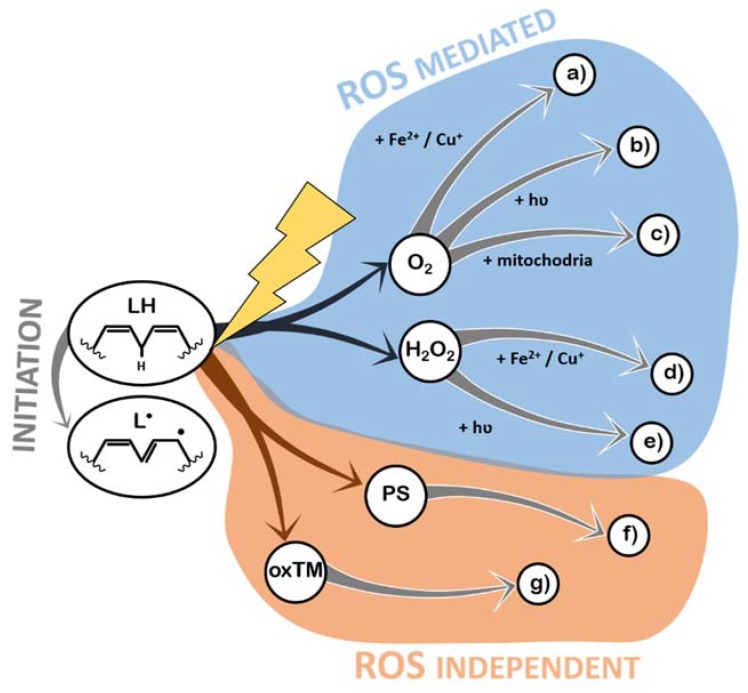
Schematic representation of lipid peroxidation initiators, according to their main features: ROS-mediated vs. ROS-independent mechanisms. PS—photosensitizer; oxTM—oxidized transition metal (Fe^3+^ or Cu^2+^). (**a**) Production of ROS (Superoxide Anion Radical, •O^2–^) by Iron (II) Fe^2+^ and Copper (I) Cu^+^; (**b**) Production of ROS (^●^O_2_^–^) by Light and Atmospheric Oxygen; (**c**) Production of ROS by Aerobic Metabolism; (**d**) Production of ROS (^●^OH) by Fenton Reaction (Reduced Metals and H_2_O_2_ Reaction); (**e**) Production of ROS (^●^OH) by Light and Hydrogen Peroxide; (**f**) Direct Oxidation by Light and a Photosensitizer (PS); (**g**) Direct oxidation by Iron(III) Fe^3+^ and Copper(II) Cu^2+^.

As with most oxidative processes, LPO is known to be caused by exposure to oxygen (because of ROS formation) and ROS formed by other chemical pathways, light, or high temperatures [31]. In this section, the detailed pathways through which each of these triggers actually induces LPO are described, in order to identify the potential targets of antioxidant systems. All the mechanisms described below can be found schematized in Figure 3. 

Indeed, exposure to ROS is the most prevalent mechanism of LPO. ROS can be generated from atmospheric oxygen in the presence of transition metals [40] (Figure 3a and Section 2.2.1) or hydrated electrons [41] (Figure 3b and Section 2.2.2). More complex enzymatic pathways related to aerobic metabolism also generate ROS [42] (Figure 3c and Section 2.2.3), as well as Reactive Nitrogen Species (RNS), under special circumstances, who have also been shown to cause LPO [43,44]. In addition, hydrogen peroxide (which naturally occurs in the atmosphere and in organic matrices) is unstable and a continuous source of ROS in the presence of transition metals [40] (Figure 3d and Section 2.2.4) or light [45] (Figure 3e and Section 2.2.5). Photo-oxidation of lipids is another form of LPO, and it is always mediated by a photosensitizer (a molecule that receives a photon and excites other molecules), which can then promote lipid radicalization directly or via singlet oxygen [31] (Figure 3f and Section 2.2.6). Transition metals, besides contributing to the generation of ROS, can react directly with lipids, leading to hydrogen abstraction and initiation of the LPO reactions [46] (Figure 3g and Section 2.2.7). Generally, the role of temperature in LPO is the acceleration of the reactions that create the lipid radicals.

#### 2.2.1. Production of ROS (Superoxide Anion Radical, •O^2–^) by Iron (II) Fe^2+^ and Copper (I) Cu^+^ (**a**)

Transition metals, like iron and copper, in their reduced states (such as Fe^2+^ and Cu^+^), can be oxidized by atmospheric triplet oxygen, to form superoxide anion radicals and Fe^3+^/Cu^2+^ [40]. Despite the fact that copper is more reactive (higher reducing potential), it is much less abundant than iron in organic matrices, in general [47]. Superoxide anion radicals have low reduction potential and cannot initiate LPO, but they can be converted to hydroperoxyl radicals under low pH, and hydroperoxyl radicals are strong oxidizers that are capable of initiating LPO [30,31,33,41]. Furthermore, Iron (III) and Copper (II) can further amplify LPO by the direct oxidation by Iron (III) Fe^3+^ and Copper (II) Cu^2+^ (Figure 3g). In Section 2.2.8, a feature common to all transition-metal-mediated LPO is highlighted. 

#### 2.2.2. Production of ROS (^●^O_2_^–^) by Light and Atmospheric Oxygen (b)

Light irradiation onto an organic, wet matrix can generate hydrated electrons, i.e., electrons that are solvated and available to react. These electrons can reduce atmospheric oxygen, generating superoxide anion radicals [41]. This mechanism has been shown to be of great relevance both in health [48] and in food quality [49].

#### 2.2.3. Production of ROS by Aerobic Metabolism (c)

Cellular respiration involves the oxidation of organic compounds so that an electron can be abstracted from these molecules and transported in a chain (the electron transport chain, ETC), until being accepted by oxygen, which is converted to water [42]. Sometimes electrons leak from the ETC before reaching the end, causing oxygen molecules to ionize (generating superoxide anion radicals) [50]. In living cells, this ROS is mitigated by Superoxide Dismutase (SOD), which converts it to H_2_O_2_. Then, catalase neutralizes H_2_O_2_ into oxygen and water. However, during their existence, both superoxide anion radical and hydrogen peroxide might induce oxidative damages (via the remaining mechanisms described).

#### 2.2.4. Production of ROS (^●^OH) by Fenton Reaction (Reduced Metals and H_2_O_2_ Reaction) (d)

Fenton reactions are a well-known set of reactions in which ROS are generated by the reduction of H_2_O_2_ to a hydroxide anion (OH^−^) and a hydroxyl radical (^●^OH) through the oxidation of transition metals [51]. Additionally, Iron (III) and Copper (II) can further amplify LPO by the direct oxidation by Iron (III) Fe^3+^ and Copper (II) Cu^2+^ (Figure 3g).

#### 2.2.5. Production of ROS (^●^OH) by Light and Hydrogen Peroxide (e)

Hydrogen peroxide itself is not a very strong oxidizer. However, as it is an unstable molecule, it can decay into different ROS by different mechanisms. Further, this molecule exists in biological systems as a product of ROS detoxification and is present in the atmosphere [31]. Upon irradiation with UV light, H_2_O_2_ is converted into two hydroxyl radicals.

#### 2.2.6. Direct Oxidation by Light and a Photosensitizer (PS) (f)

Molecules that absorb light are capable of transferring the photon’s energy to other molecules (photosensitizers) and can initiate LPO if light excites them under low-oxygen-concentration conditions. In these cases, if enough energy was transferred to the photosensitizer (chlorophylls and hemoglobin, among others), hydrogen abstraction occurs from lipids directly [52]. If, however, oxygen is abundant, then it is the most likely receiver of the energy contained in the photosensitized molecule, generating singlet oxygen from triplet oxygen. This form of excited oxygen has been shown to initiate LPO by directly interacting with double bonds in lipids [52], creating unique peroxidation lipid by-products.

#### 2.2.7. Direct Oxidation by Iron(III) Fe^3+^ and Copper(II) Cu^2+^ (g)

The more oxidized forms of iron and copper (trivalent and divalent cations, respectively) have a sufficiently high reduction potential to cause a proton to abstract from a methylene group [46], stealing an electron (being reduced into Fe^2+^ or Cu^+^), and leaving a lipid radical as result. 

#### 2.2.8. Oxidative Triggers Cyclic Regeneration

**Figure 4:**
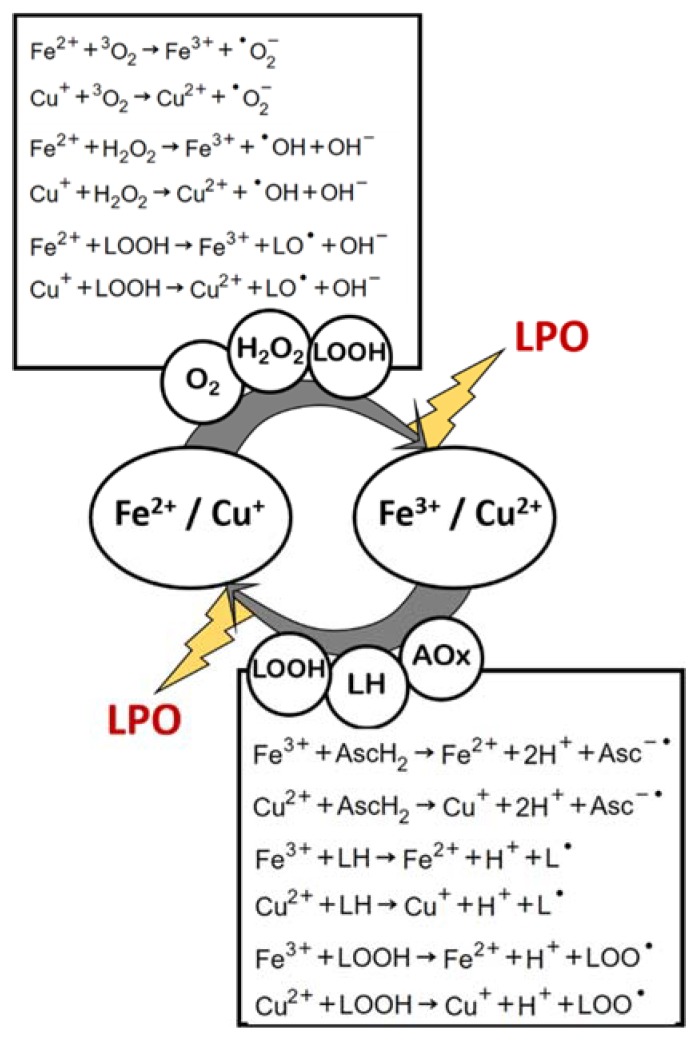
Transition metals (iron and copper) redox recycling and its relevance in lipid peroxidation (LPO). AOx: Antioxidant.

It is noteworthy that the product of two of the mechanisms here described (a and d) is the reagent in mechanism g, and the product in mechanism g is the reagent in mechanisms a and d. This leads to an amplification of LPO by means of a redox cycle that constantly regenerates oxidative triggers, and such regeneration is in itself an LPO inducing reaction (Figure 4). This recycling of transition-metal-induced LPO initiation potential is further augmented by other parallel, yet very much possible reactions, in a biologic or nonliving organic matrix: (i) the reactions of Fe^3+^ or Cu^2+^ with certain antioxidant compounds (which become pro-oxidants in this case), such as ascorbic acid [31,53,54,55]; and (ii) the reactions of iron and copper cations (whether in their more reduced or oxidized forms) with lipid peroxide, yielding lipid alkoxyl- or peroxyl-radicals [46,56] (Figure 4). 

### 2.3. On the Quantification of Lipid Peroxidation

Several methods to assess LPO have been used by researchers and clinicians, and have been extensively reviewed elsewhere [30,39]. Along the process, the physicochemical changes associated with undergoing and/or terminated LPO can be used by researchers to measure the extent of the process. As seen above, oxygen is consumed during LPO, and oxygen consumption rate has been used as a measure of LPO [57,58]. Evidently, this method detects LPO indirectly and is a weak proxy for the process, as oxygen consumption by other oxidative processes, by respiration of biological nature, solubility changes, and others, may influence the results. As discussed in the previous sections, upon undergoing oxidation, the originally present lipid (LH) suffers several structural changes leading to different forms, including L^●^, LOO^●^, LOOH, and LO^●^, as well as lipid adducts, such as LOOL, LL, or LR, being R any of a series of biomolecules, including DNA and proteins. Each of these products has been used to monitor lipid peroxidation [39,59]. However, these species are either short-lived or not guaranteed to occur, depending on the matrix and oxidative conditions. Besides, Liquid Chromatography coupled to Mass Spectrometry (LC-MS) techniques used to quantify these products are expensive and time-consuming, and so are immune-based assays or High Performance Liquid Chromatography coupled to chemiluminescence (HPLC-CL) based ones. The spectrophotometric detection of conjugated dienes, however, has been shown to be a rapid, low-cost method to monitor LPO, especially if used to analyze samples undergoing LPO along time [60]. Upon oxidative stress, and having set the baseline absorbance at 234 nm, the change (increment) of absorbance at this wavelength is directly proportional to the formation of lipid peroxyl radicals.

Another approach to LPO quantification is the use of secondary end-products as indicators of the extent of the reaction. Some methods have been described for the detection of pentane, ethane, or HNE; however, their relevance is essentially in a clinical context, for analysis of human-derived samples [39,59,61,62]. Regardless, and by far, LPO has been mainly quantified by the amount of MDA in the samples. This is because an easy, low-cost methodology for the spectrophotometric detection of MDA exists: the measurement of thiobarbituric acid reactive species (TBARS) formation upon reaction with TBA [59,63,64]. MDA forms adducts with TBA that strongly absorb light at 532 nm. This reaction has been exhaustively used in the literature to quantify MDA, assuming it to be an indicator of LPO. As with most spectrophotometric methods, however, TBARS assay has been shown to be unspecific [65], given its potential to generate false positives (by TBA reacting with aldehydes other than MDA). Other methods have been suggested to quantify MDA (such as isolation and quantification by HPLC prior to reaction with TBA), but they are evidently not as practical, and TBARS remains the gold standard of MDA quantification (and, therefore, of LPO quantification). However, as with most indirect methods (that use secondary end-products as estimators of a given reaction), MDA quantification in itself has been shown to be an unreliable measure of LPO, since MDA is a product of reactions other than LPO [66] and is also very reactive: Depending on the composition of the matrix and conditions of the sample, it will be consumed in the chemical reaction [67]. Nonetheless, TBARS has been one of the most used methods to address the development of LPO in the in vitro models reviewed below.

For in vitro systems where no chemical oxidizing agent is added, but LPO occurs through different pathways, lipid peroxides thus formed become the main oxidizing compounds in the medium, and LPO can be quantified by using a reagent prone to be oxidized by lipid peroxides (amount of reagent lost can be converted in amount of lipid peroxides). This is the case of the thiocyanate method [68], which has been used to track LPO in lipid-solution-based LPIP assays (as described in the next section) [69,70,71,72,73,74,75,76,77,78]. Given the lack of an oxidizing trigger in these assays, the capacity of the medium to oxidize iron (II) to iron (III) is directly proportional to the concentration of lipid peroxides. Importantly, one should note that quantifying the lipid peroxides of a sample is a measure of LPO that will decay along time, as lipid peroxides degrade into smaller molecules; thus, it is a measure of “early lipid peroxidation”. In the thiocyanate method, ferrous ions are oxidized into ferric ions, which in turn form a colored complex with thiocyanate (ferric thiocyanate), with maximum absorbance at 485 nm [68,69,70,75,76,77].

Another “early lipid peroxidation” measure that can be obtained in real time is the chemiluminescence associated to LPO, i.e., the emission of photons by chemical species that are present when LPO is occurring. Different types of photons can be emitted as a consequence of LPO, and used to track this reaction [79], depending on the source molecule. However, the most used chemiluminescent methods for LPO detection, relevant in the field of LPIP assays, are the detection of lipid hydroperoxides by HPLC-CL [80,81,82] and the detection of tert-butyl hydroperoxide (t-BOOH)-initiated chemiluminescence [83,84,85,86]. In the case of lipid hydroperoxide detection by HPLC-CL, eluted lipid hydroperoxides are mixed with a chemiluminescence initiator reagent (such as cytochrome c—luminol mixtures, or microperoxidase–iso-luminol mixtures) [80,81] and a chemiluminescence detector is used to quantify photon emission. For t-BOOH initiated CL, t-BOOH is added to the lipid substrate as a radical generator [83,84,85,86], which, in turn, produces lipid peroxyl and alkoxyl radicals responsible for the excitation of triplet oxygen into singlet oxygen and the generation of excited state carbonyl small molecules, both responsible for emissions of photons [79].

## 3. In Vitro Quantification of Natural Products Lipid Peroxidation Inhibitory Potential

LPO quantification has been extensively used in research and can be achieved by a variety of methodologies. However, it has found its main application in the field of physiology and toxicology, as a biomarker of oxidative stress, in which cases samples are obtained from biological systems, adequately conserved to stop peroxidation, and submitted to such quantification to determine the level of LPO at the endpoint of the corresponding experimental design. In these cases, LPO occurs under in vivo conditions, where lipid substrate, oxidative triggers, and endogenous antioxidant systems are all being actively regulated by metabolic processes. In addition, in the field of product quality and safety control, lipid-rich matrices that have been under optimal conditions of packaging and storage are assayed for LPO as a routine parameter indicative of degradation. Under these circumstances, the only methodological considerations rely upon the choice of the method used in the quantification of LPO (as discussed in Section 2) and the adequate correlation of its results with meaningful lipid peroxidation measures. 

The quantification of LPO inhibition can be seen as a subtype of the assay previously described. An in vivo system (or final product formulation) subjected to natural or artificially created oxidative stress, with and without the candidate NPs, for a given time, under a set of defined environmental conditions, is the ideal model to test the LPIP of such NPs. Thus, for example, upon attempting to discover a NP with LPIP for inclusion in a functional food, to reduce LPO in the tissues of the consumer, trials with humans fed with the ingredient-containing formula versus a control group (fed with the non-modified formula) would be the ideal experimental design. In addition, for application as a preservative additive in LPO-prone food matrices, the best assay to determine the LPIP of a given NP would be to have the food product (with and without such NP as an ingredient) exposed to regular or accelerated shelf-life trials and compare the LPO levels at the relevant endpoints. 

However, in the field of NPs biodiscovery, the high number of experimental conditions (different biomass sources, biomass processing conditions, extraction conditions, extract fractionation, extract, and/or molecule application, and the different variables required to characterize the antioxidant activity, namely the time-course and dose-dependent response of substrates to oxidative degradation) make such type of experimental designs unfeasible. Here, like in many other fields of bioprospecting, the concept of screening assays (assays where there has been simplification and miniaturization of the procedure, maintaining as much representativeness of real-scale phenomena as possible) becomes of uttermost importance. Only using such platforms can high-throughput analysis of hundreds of candidates be performed, which is important given the relatively low rate of successful hits. 

All in vitro systems to study lipid peroxidation consist of a lipid substrate dispersed in a liquid media, to which an oxidizing agent is added. Then, a measure of LPO extent is obtained after a certain incubation period. Some of these steps have important nuances: Is the addition of the oxidizer a trigger of LPO that is responsible only for the initiation process? Or does its presence in the reactional media represent a constant source of radicals, contributing to the lipid damage throughout the entire incubation period? Can LPO be measured without disturbing the system, allowing easy time-dependence study, or does it require sampling and stopping the reaction proceeding to LPO measurement? Finally, appropriate treatment of experimental data to calculate meaningful values of inhibition, rates, and other indices needs to be performed. These are the subjects discussed in the following sections, which are divided based on the substrate used. The common basis of all the substrates is the content in PUFAs (free or esterified) and sterols, which are the two lipid types more prone to peroxidation. Furthermore, because lipids in organisms are integrated in a water-based environment, in the form of supramolecular arrangements, such as lipid bilayer membranes, it is natural that most methods of in vitro LPO simulation use micellar systems to co-expose the lipids and the oxidative stress. Even in nonliving organic matrices, like foods, feeds, and cosmetics, water is abundant and lipids are stabilized by other organic compounds, maintaining them dispersed, in solution. 

### 3.1. Rat Liver/Brain-Based Assays

Based on the LPO in vitro model being used at the time for physiological studies on LPO (see the works of Wills et al. [87,88] and the works of Kornbrust et al. [89,90]), many studies looking for the LPIP of molecules or extracts started by using rat liver microsomes as substrate. Building upon the use of rat liver and rat brain as reference tissues to produce this type of lipid-rich micellar systems, most studies now perform the LPO assays by using the whole tissue homogenates as substrate/reactional medium. Some of the first works of LPIP determination of NPs using rat liver/brain homogenates and derivatives can be found in the literature and are herein suggested [83,84,85,86,91,92,93,94,95,96,97]. Noteworthy, to these days, works using rat liver/brain-based assays still reference these older articles and follow their methodologies for the preparation of the substrate. Interestingly, a tendency shift from the use of microsomes to the use of whole tissue homogenates has been found, as well as a tendency shift from liver to brain tissue usage, partly justified by an increased interest in neuroprotection against oxidative stress.

Preparation of rat liver or brain homogenates, as well as fractions of these (mitochondria and microsomes), follows a general procedure in all the works. Briefly, lab-grown rats are sacrificed, and their livers and brains are excised. Mechanical homogenization is then performed, typically in a biological buffer, and a low-speed centrifugation is used to discard insoluble solids. The supernatant is a solution containing the soluble compounds of the tissues, as well as small particles, such as mitochondria and microsomes. This solution is often used as-is in the LPIP assays. However, further isolation of especially LPO-related fractions of this solution (the mitochondria, due to increased ROS concentration, and the microsomes, due to elevated lipid and redox enzymes contents) can be obtained by differential centrifugation. Usual buffer compositions used in the homogenization of rat tissues, as well as centrifugation conditions for the isolation of fractions, are summarized in Table 1.

One of the possible arguments for using these substrates is the fact that they contain a high amount of lipids from the biological membranes, while containing the other key players in the oxidative phenomena that occur in cells, such as the enzymes and metal ions, in concentrations that are comparable to those in vivo. Thus, the system provides a complete reactional media in which LPO would occur at conditions similar to those found in the corresponding living system. However, because this method for LPIP determination was built using the methodology followed by researchers that were testing the effect of different oxidizers in LPO, nowadays different approaches to induce oxidation in this system can be found. Some of these approaches make more sense than others, from the biochemical point of view, but ultimately the goal is to be able to create an increase in measurable LPO in order to compare controls and samples containing the candidate NPs. Table 2 summarizes the conditions for LPIP determination used in selected works performed with these rat-derived substrates.

As can be seen (Table 2), different approaches have been developed deriving from the use of rat liver or brain homogenates and derivatives, such as microsomes. Differences between oxidation triggers greatly influence the results found, as different initiation mechanisms might be occurring, thus detecting LPIP from different mechanisms of antioxidant action. Moreover, differences in the oxidation triggers result in incubation times and concentration ranges for the reagents that require optimization to result in measurable and useful LPO values (above limit of detection and below plateau at maximum oxidation). Additionally, the use of homogenates and supramolecular arrangements, such as microsomes, leads to increased turbidity, which might create difficulties in regard to spectrophotometric readings.

Overall, studies in Table 2 demonstrate the variety of methods to induce oxidation that have been used; considering the fact that lipid peroxidation is a propagative reaction, it seems odd that most studies reviewed choose to use metal-catalyzed oxidation systems as triggers, which self-regenerate (actually, ascorbic acid has been purposefully added in some cases, contributing to a cycling of oxidation states of these metals). This may represent a problem, since after initiation, two rates of formation of lipid peroxidation products will co-occur and will vary differently in time, resulting in a nonlinear, multifactorial response. Concerning this choice of trigger, two important notes must be added to this discussion: (a) that different combinations of transition metals in different oxidation states, with different forms of redox catalyzing agents in different states of protonation (such as ascorbic acid, ascorbate and dehydroascorbate), sometimes with the addition of H_2_O_2_, have been used, each of them initiating LPO by a different mechanism (see previous chapter), being very difficult to compare; and (b) that some of these systems work by producing ROS (those based on higher concentrations of reduced metals, Fe^2+^ and Cu^+^, with or without H_2_O_2_), while others may induce peroxidation without ROS as intermediates (those with higher concentrations of oxidized metals, Fe^3+^ and Cu^2+^), and therefore the determined LPIP will be highly influenced, for example, by the capacity of the NP to scavenge ROS or to chelate metal ions, respectively—due to neutralization of the initiator.

Furthermore, the prevalence of studies basing their LPIP evaluation at a certain time-point measurement of TBARS might constitute a potential problem, since a reproducible LPIP value is only possible to obtain in a certain temporal window of the kinetics of LPO with and without antioxidant: at the beginning of a plateau in LPO by-products in the control reaction. By skipping the characterization of a time-dependent curve of MDA formation, these studies might be measuring LPO too soon (both control and treatment samples have low values, because lipid peroxides have not yet been cleaved, resulting in the underestimation of LPIP) or too late (both treatment and control samples have reached the maximum LPO, determined by substrate concentration, given the propagation reactions and the aerobic conditions; eventually, every lipid will be oxidized, and inhibition cannot be measured).

Lipid peroxidation initiated by t-butanol hydroperoxide (t-BOOH) may provide a more reproducible protocol to determine an NP’s LPIP, despite overlooking the LPIP indirect component. The addition of a finite concentration of t-BOOH to a lipid substrate will serve as a reproducible initiator, which will not be regenerated, and therefore a clear initiation rate (transference of the peroxide from butanol to the lipid) and propagation rate can be calculated; these rates can then be used to determine different parameters of LPIP (kinetics-related). The studies reviewed dealing with t-BOOH-initiated LPO detected the reaction by using chemiluminescence, which is a rather specific method, but requires equipment that is not very common in most labs.

Despite the wide use of this substrate, resulting in an abundance of results from its application, it presents several disadvantages concerning the development of a fast, easy screening protocol for novel antioxidant discovery: First, its preparation is laborious, cost-intensive, and ethically controversial (it requires controlled-conditions-grown animals, sacrifice, tissue homogenization, and eventually a series of laborious centrifugation steps); second, the variability between batches concerning its natural content in antioxidant enzymes, antioxidant organic compounds, pro-oxidant enzymes, pro-oxidant organic compounds, and different oxidation state metal ions represents a source of variability concerning initiation, incubation, and detection protocols, thus requiring optimization or dose- and time-dependent studies; third, the presence of biological molecules such as proteins leads to a constant loss of MDA and other small LPO by-products to secondary reactions, worsening the problem of reliability in detection methods, such as TBARS.

Upon reviewing the efficacy of the substrates that are covered below, it becomes apparent that using microsomes and tissue homogenates as substrate in an in vitro LPO model to test antioxidants is particularly useful in the case of screening for LPO inhibitors via inhibition of enzyme-mediated LPO, while the screening of NPs with good LPIP regardless of the mechanism is probably better performed by using less complex, more batch-independent (thus, reproducible) matrices.

### 3.2. Low-Density Lipoprotein-Based Assays

A low-density lipoprotein (LDL) is a complex of peptidic and lipophilic compounds that occurs in blood from different animal species, and its function is to transport lipids across the body [112]. One of the reasons LDL particles have been used in developing in vitro lipid systems to test peroxidation is exactly the fact that these particles successfully solubilize fats in aqueous media, which is the main reason for their chemical configuration in vivo. Another reason for an augmented interest in using LDL-based assays of lipid peroxidation is the fact that, in vivo LDL, particles’ peroxidation is of extreme biological relevance [112]. Oxidative damage to LDL has been correlated with several pathologies, and testing antioxidants directly in LDL, even though in vitro, may provide a more meaningful insight on the potential of NPs to promote in vivo LDL protection. LDL particles used in LPIP assays herein reviewed (Table 3) were isolated from human blood samples, with the exception of one study that used rat blood instead [113].

The procedure of LDL recovery was very similar between works: Human blood is collected from normolipidemic, healthy, fasting volunteers and centrifuged to isolate plasma. Plasma thus collected, or commercially obtained, is then subjected to widely validated ultracentrifugation methodologies, commonly used in the fractionation of blood lipoproteins. Because the goal of these LDL particles is to be used in oxidation assays, dialysis of the LDL fraction obtained by centrifugation has been frequently performed [114,115,116,117,118,119], to eliminate metal ions and other contaminating pro-oxidants. For storage purposes, it has been common to solubilize the LDL particles in a buffer containing EDTA [116,117,120], and sometimes an additional antioxidant protection (e.g., BHT) [60]. Thus, before the oxidation assay, repeating dialysis may be necessary to remove these protectors and re-obtain LDL particles susceptible to the oxidative trigger used [116]. Resuspension of LDL particles for the assay is usually performed in either saline solution or PBS (pH 7.4) that is often subjected to oxygenation [60,121,122].

An overview of oxidative triggers’ nature and concentration can be found in Table 3, along with substrate concentration and detection and incubation approaches. The LDL-based systems used to determine LPIP have been essentially based on the oxidation of LDL by transition metals, namely copper (II), in the presence of oxygen. Similar to what happens in the protocols using rat liver and brain derivatives, to the best of knowledge, the reason to choose this self-regenerating system is unclear. However, the protocol seems to be better established and more homogeneous when using LDL substrate. Besides, several of these studies have performed time-dependent curves of LPO for different concentrations of substrate and copper (II), as measured by conjugated dienes concentration estimated from absorbance at 234 nm, which provides a solid literature basis to define endpoints, timescales, and concentration ranges to work with.

**Table 3:** Overview of assay conditions used in models with low-density lipoprotein as substrate; ns—not stated; CD—conjugated dienes.

Oxidation Trigger		LDL Concentration	Incubation and Detection	Ref.
Type	Concentration			
Cu^2+^	1.66 µM	0.25 mg/mL	CD, TBARS and iodometry along time (Room Temperature)	[60]
	10 µM	50 μg protein/mL	CD along time (37 °C); TBARS (2 h at 37 °C)	[118]
	3 µM	100 μg protein/mL	TBARS and electrophoretic mobility after 6 and 24 h at 37 °C	[117]
	5 µM	0.3 mM cholesterol	CD along time; TBARS and electrophoretic mobility after 4 and 24 h at 37 °C	[123]
	ns	0.08 mg cholesterol/mL	CD along time at 30 °C	[122]
	ns	1 mg protein/mL	TBARS after overnight at 37 °C	[124]
	1 mM	150 ug/mL	CD along time at 37 °C	[120]
	20 mM	150 ug/mL	TBARS after 3 h at 37 °C	[120]
	5 µM	100 μg protein/mL	TBARS after 90 min at 37 °C	[116]
	5 µM	0.1 uM	CD along time at 37 °C	[121]
	0.1 mM	200 μg protein/mL	CD along time at 37 °C	[125]
Cu^2+^/H_2_O_2_	40 µM/80 µM	3.15 mg protein/mL	Electrophoretic mobility after 3 h at 37 °C	[105]
AAPH	4 mM	0.3 mM cholesterol	CD along time; TBARS and electrophoretic mobility after 4 and 24 h at 37 °C	[123]
	5 mM	200 μg protein/mL	Cholesteryl esters hydroperoxides quantification by HPLC-UV along time at 37 °C	[115]

LDL oxidation for the purpose of in vitro determination of LPIP has also been performed by using an organic radical generator—2,2′-azobis (2-amidinopropane) dihydrochloride (AAPH)—as initiator, which is a much more controllable, reproducible manner of inducing oxidation than transition metals [115,123]. In addition, these authors performed the studies by using reliable detection methodologies along time, allowing further method development works to build on this knowledge. The increased simplicity of the reactional medium thus obtained may facilitate miniaturization and increased throughput of LPIP assays to be developed.

Because LDL particles have three-dimensionally packed lipids and proteins—which is why they are soluble—different lipophilicity of the NPs being tested may affect the results of LPIP via different concentrations of each component of the NP in the microenvironment of the lipids. For this reason, we believe further studies should determine whether a preincubation of the substrate and sample, before adding the initiator, is beneficial to promote the equilibrium of partition of the compounds between the solvent and LDL particles.

### 3.3. Lipid Solutions Based Assays

#### 3.3.1. Linoleic Acid Micelles and Emulsions and β-Carotene Bleaching Assay

Pure linoleic acid dispersed in an aqueous media is probably the simplest substrate that can be used for LPIP in vitro determination. However, because solubility of linoleic acid in water is low, the substrate preparation should be carefully considered when using this system. Despite being the same molecule, systems of linoleic acid below its critical micellar concentration (CMC), above the CMC, and in the presence of surfactants (generating an emulsion) will fundamentally result in different assays. This is because the distribution and efficacy of different lipophilicity NPs will be affected by the state of solvation of the substrate. For example, in an emulsion, the oil-phase may become enriched in more lipophilic molecules, such as carotenoids, while phenolic compounds may remain in the aqueous phase. Both types of NPs will be found simultaneously in most ethanol or acetone extracts of plant material. Then, the same extract will probably display different LPIP values at the same concentration, depending on the size of oil droplets of the emulsion, which, in turn, will depend on the method of preparation of the substrate. Table 4 compiles some linoleic acid dispersion methods available in the most recent literature using this substrate for LPIP determination.

Different methods available to disperse linoleic acid in a medium that is compatible with oxidizing conditions allow the adaptation of the medium for NP dissolution. For instance, the work of Freitas et al. [76] used a system mostly composed of ethanol, which might allow the LPIP determination of a wider range of NPs (including some less polar extracts) using water-soluble radical initiators (such as AAPH) or transition metals. In more aqueous systems, the use of saponified linoleic acid (the sodium salt, for instance) to increase water solubility, with [70,73,128] or without [70,126] the addition of a surfactant, might be a solution to test very hydrophilic compounds (such as natural polysaccharides), which are insoluble, even at low concentrations of organic solvents.

Moreover, because linoleic acid is extremely prone to LPO, even without the presence of metal ions or other catalysts (only atmospheric oxygen and/or light), there is no need to induce oxidation by using a chemical component with an oxidizing potential [129]. Thus, lipid peroxidation can be detected by using methods that rely on oxidation themselves, since the lipid peroxides will act on the reagents and cause their oxidative state to increase, developing color. For this reason, linoleic-acid-based systems have often been used as LPO quantification method in the thiocyanate–iron(III) complex spectrophotometric assay [69,70,71,72,73,74,75,76,77,78], which is an advantage of using this substrate given the increased simplicity and decreased interferences of this method.

A derivative of linoleic-acid-based systems of LPO simulation used for LPIP determination is the β-carotene bleaching assay. Effectively, this is the assay that has gained more popularity as an LPIP assay and has found application in many works dealing with antioxidant capacity of NPs. In this method, the reactional media is like those described above (3.3.1), but contains β-carotene, a colored compound that is highly reactive with peroxidized linoleic acid, and which loses color alongside such reaction. Thus, spectrophotometric variation of the solution’s color (a decrease in absorbance at approximately 470 nm) is representative of the generation of lipid peroxides. The procedure, advantages, and disadvantages of this system have been extensively discussed in the literature [130,131,132,133,134,135,136,137,138], and some examples of the latest LPIP research employing this method are suggested [139,140,141,142,143,144,145,146,147,148,149].

Overall, despite being widely recognized as an LPIP assay, this method has been shown to lack reproducibility, mainly due to the complexity and variability associated to the reagent preparation [131]. Besides, because oxidizing agents would cause very rapid color loss by directly oxidizing β-carotene, LPO is often induced by heat (around 50 °C), which is a non-specific mechanism of oxidation (actually, it only increases the rate at which oxidation is occurring, e.g., due to atmospheric oxygen) [132,133], being the time-response of NPs antioxidant activity and control highly dependent on system characteristics, such as pH, contaminants, and solvent composition [134,138]—the latter being a problem since chloroform is very volatile, and its concentration in the reactional medium is hard to maintain stable. Furthermore, β-carotene’s direct degradation due to light, oxygen, and heat has been shown to have an impact in LPIP determinations [130,133]. Modifications of the original method [136] have attempted to fix the operational difficulty (reagent simplification) [131,135], as well as the miniaturization [131,137], and improvement of data interpretation [137].

Since many NPs are tested in the form of crude extracts, and most originate from plant sources, it is natural that carotenoid pigments confer color to natural extracts. The contribution of the extracts color itself is therefore a bias, for which correction by the addition of sample blanks (reactions containing only the extract and no substrate) is not reliable, since the extracts color itself is prone to modification during the time of the assay. Thus, the β-carotene bleaching assay is more reliable when determining LPIP in isolated, colorless NPs.

#### 3.3.2. Phospholipid Micelles and Liposomes

In an attempt to decrease the complexity and interferences of in vitro systems using biological derivatives as substrates, which contain protein, metals, and other components that may influence oxidation reactions, while maintaining some representativeness of biological systems, a model of phospholipid bilayer membranes (which are easily solvated in water), called liposomes, was used to estimate LPIP [69,150,151,152]. Because liposomes have applications in the drug delivery field, and their study is used by researchers interested in cell membrane properties, several procedures for their preparation are described in the literature that are far more complex than required for a LPIP assay, in which homogenous lipid distribution and constant contact area with oxidation triggers during the assay are the main factors affecting the assay reliability. Thus, a compilation of works dealing with liposome preparation, using different polar lipids with the purpose of LPIP, is presented in Table 5, as well as the conditions of induction, incubation, and detection.

Liposomes are usually prepared by dissolving polar lipids in organic solvent, evaporating the solvent (using a stream of gaseous nitrogen instead of rotary evaporation has been suggested to reduce initial peroxidation values [153,154,155]), and then resuspending them in aqueous media, using vigorous agitation and sonication, to ensure hydration of the lipid film and reduction in vesicle size and number of lamellae [156]. Alternative/complementary steps exist, such as freeze–thaw cycles using liquid nitrogen [73] or the use of nanopore filters to extrude the liposomes multiple times [153,154]. However, more complex protocols are useful in the homogenization of particle size and number of lamellae distributions, while simpler sonication-only dispersions of polar lipids might be enough for LPIP determination [157].

Because of the packing of lipid molecules with polar heads toward the interior and exterior of the vesicles, and the aliphatic chains of each layer toward each other, liposome systems may allow the intercalation of lipophilic antioxidants in the evaporation phase and the occurrence of hydrophilic antioxidants in both sides of the membrane, in the aqueous media [155,158,159]. This is an important feature, as it is known that, in biological systems, lipophilic antioxidants are often found in the inner part of phospholipid bilayers, to offer chain-breaking protection against LPO. Similarly, the capacity of hydrophilic antioxidants to pass through liposomes pores to the inside of the liposomes after their formation might simulate the capacity of that NP to enter and protect cells against intracellular oxidative stress. The advantages of liposome models, however, can only be obtained if liposome preparation is performed in very specific conditions and their structural qualities are determined after production [156]. For this reason, liposomes represent a more laborious and expensive method to screen NPs’ LPIP. As a final note, Sowmya et al. [155] described a liposome-based method to assess singlet oxygen-initiated peroxidation, which uses methylene blue as a photosensitizer. This is an important addition to the panel of oxidation triggers herein discussed, as, more commonly, in vitro simulations of LPO have dealt with metal or radical mediated mechanisms of action.

**Table 5:** Overview of assay conditions used in models with liposomes as substrate. PL—polar lipid; ns—not stated; AscH^−^—ascorbate anion; AscH_2_—ascorbic acid; NP—natural product; CD—conjugated dienes; PL-OOH—phospholipid hydroperoxides.

Substrate Preparation		Substrate Concentration (mg/mL)	Oxidative Trigger		Incubation and Detection	Ref.
Polar Lipid	Liposome Generation		Type	Concentration		
Mix of Bovine Brain Phospholipids	PL in CCl_3_ (1 mg/mL) Vacuum evaporationResuspen-sion (1 mg/mL) in NaCl 0.9% Ultrasounds 15 min	0.3	Cu^2+^	5 µM	Endpoint measurement by TBARS and fatty acid profiling after 24 h at 37 °C w/ atm O^2^ and light	[160]
Soya Bean Lecithin	Suspension of PL in water (8 mg/mL) Ultrasounds 30 min (20 KHz)	7.9	Cu^2+^	20 µM	CD and TBARS over time at 31 °C with agitation	[74]
Lecithin	PL in CCl_3_:Meth-nol 86:14 (10 mg/mL) N_2_ (g) evaporation Resuspen-sion (5.6 mg/mL) in warm PBS Ultrasounds 60 min (on ice)	3.6	AAPH	10 mM	Endpoint measurement by TBARS after 2 h at 37 °C	[161]
Soybean Phosphatidyl-choline	PL in CCl_3_ (10 mg/mL) N_2_ (g) evaporation Resuspen-sion (5 mg/mL) in His-buffer (5 mM, with 0.12 M KCl pH 6.8) 37 °C /1 h/agitation 10x extrusion through 2-stacked 100 nm pore polycarbonate filters	0.1	Fe^3+^/AscH^−^	50 µM/100 µM	Endpoint measurement by TBARS after 1h at 37 °C	[153,154]
Egg yolk phospholipids	PL in CCl_3_ (2.5 mg/mL) + sample in methanol N_2_ (g) evaporation Resuspen-sion (0.625 mg/mL) in water Ultrasounds 5 min (50 Hz)	0.625	Light	ns	CD along time at 50 °C	[155]
Egg yolk phospholipids	PL in CCl_3_ (2.5 mg/mL) + sample in methanol N_2_ (g) evaporation Resuspen-sion (0.625 mg/mL) in Methylene Blue aqueous solution (50 µM) Ultrasounds 5 min (50 Hz)	0.625	Light + Singlet Oxygen	ns	CD along time at 50 °C	[155]
Egg lecithin	PL in PB (10 mM pH 7.4) (10 mg/mL) Ultrasounds 30 min	9.5	Fe^3+^/AscH_2_	6.3 µM/6.3 µM	Endpoint measurement by TBARS after 1 h at 37 °C	[157]
Egg phosphati-dilcholine	PL and sample in CCl_3_:Metha-nol N_2_ (g) evaporation Resuspen-sion in Tris-HCl (10 nM pH 7.4, 0.5 mM DTPA) Vortex Ultrasounds 30 s 21x extrusion through 100 nm pore polycarbon membrane	ns	AAPH	0.5 mM	PL-OOH along time by HPLC-UV	[152]

## 4. Conclusions

Lipid peroxidation is a relevant chemical phenomenon for society, given its consequences in human and animal health, as well as its economic impact deriving from the spoilage of lipid-rich foods, feeds, and cosmetic products, among others. Therefore, a great interest in the development of technologies—among which, novel natural products—capable of inhibiting this reaction in both living and nonliving organic matrices is clearly the subject of much interest by researchers and industrial stakeholders. Similar to other activities found through bioprospecting, the development of a method that is reproducible and standardized to evaluate natural products’ capacity to protect lipids against oxidative degradation is of great importance. Such a method should also be compatible with the high-throughput natural product discovery. By reviewing the literature on LPIP assays, it was possible to detect that several strategies have been used to assess LPIP of NPs in vitro, using different combinations of lipid substrates and oxidizing agents.

Concerning the substrate, most LPIP studies have used biological derivatives as substrates, namely rat tissue homogenates, rat tissue microsomes, and human blood LDL particles. The advantages of these methods have been identified as being mostly related to the representativeness of these complex media concerning actual in vivo conditions—namely, the affinity of the antioxidant molecules to the microenvironment of the substrate phase, which models the actual antioxidant activity, and in the case of microsomes, the presence of enzymes from the natural antioxidant systems of living organisms. However, because these substrates have been isolated from their living environment, such representativeness requires validation that has not been yet performed, while the disadvantages of using such substrates (the labor-intensiveness of obtaining them and the potential variability between studies) question their usefulness. Moreover, studies using lipid solutions as substrate, either in the form of micelles, emulsions, or liposomes, have also been performed and do not present the problem of variability and labor-intensiveness, and for that reason might be preferable for screening purposes.

Oxidizing conditions used to promote these lipidic substrates peroxidation, to determine NPs’ LPIP, have also been of very different natures. As reviewed here, there are several pathways of initiation depending on atmospheric oxygen, light, and transition metals, among others, and the effectiveness of an antioxidant, as well as the type of by-products that can be obtained using each one, will suffer relevant variations depending on these pathways. Thus, the fact that LPIP has been screened by using so many different oxidizing approaches makes it very clear that results present in the literature of LPIP are hardly comparable, and sometimes of little biochemical significance (e.g., when a redox cycling oxidant is used and LPIP determined too late, no antioxidant could ever present a good value of LPIP). Finally, the choice of the detection methodology and incubation period has often been performed either by partially copying the existing literature—which may present a problem, since none of the source protocols had been validated concerning time and dose-dependent kinetics—or by a preliminary optimization that has not been reported in most studies, leading to difficulty in reproducing the assays.

Of no less relevance is the fact that, to the present knowledge, only one study [137] presented a protocol miniaturized to 96-well microplates. Even this study failed to validate the method by properly characterizing the kinetics of lipid peroxidation with substrate and oxidizing agent concentrations, as well as through time. Natural products screening often requires high-throughput analysis, and reduced amounts of samples are often available for many different assays, which makes most of the protocols here reviewed hard to use for this purpose. For that reason, even when lipids are the main substrate intended to be protected in a product or in an organism, non-specific antioxidant assays have been employed in the initial stages of NP biodiscovery (e.g., DPPH, FRAP, and/or ORAC). Evidently, it would be of great importance to establish a screening-friendly LPIP assay that could be used to narrow down candidate NPs for lipid protection against oxidation, without compromising the probability of successful hits.

In sum, it is apparent from this literature revision that a careful choice of the combination of substrate, oxidizing agent, incubation time, endpoints, and detection method has to be done during the conceptualization of the assay. Thus, a theoretical understanding of the lipid peroxidation reactions and time-dependence data of the reactions, with different types and doses of each of these elements (when applicable), need to be considered together. Preference shall be given to methods requiring the simpler and cheaper reagents, the lower number of steps, the lower volumes of reactional medium required, and the higher robustness against natural products’ associated artifacts (e.g., native color of natural extracts), as these traits make such LPIP assay suited for the NP-screening pipeline.

## Figures and Tables

**Table 1:** Homogenization solutions, biomass-to-solvent percentages, and centrifugation conditions in the preparation of rat-derived substrates used in in vitro LPO models. Unless stated otherwise, the supernatant obtained in the differential centrifugation is the substrate used; ns—not stated; HEPES: 4-(2-hydroxyethyl)-1-piperazineethanesulfonic acid.

Homogenization Solution	Biomass (% *w/v*)	Differential Centrifugation	Ref.
Rat liver homogenate			
Tris-HCl buffer (40 mM, pH 7.4)	25	ns	[94]
Phosphate buffer (50 mM, pH 7.4) with KCl 120 mM	10	700× *g*/10 min	[83,84,85,86]
Tris-HCl buffer (100 mM, pH 7.4)	10	1400× *g*/10 min	[98]
Phosphate buffer (50 mM, pH 7.4)	20	800× *g*/15 min	[99]
Phosphate buffer (100 mM, pH 7.4) with KCl 150 mM	10	800× *g*/15 min	[100]
HEPES buffer (100 mM, pH 7.2) with KCl 125 mM	25	400 rpm/15 min	[101]
Tris-HCl buffer (10 mM, pH 7.4)	10	3600× *g*/10 min	[102]
Rat brain homogenate			
Phosphate buffer (50 mM, pH 7.4)	20	800× *g*/15 min	[99]
Tris-HCl (10 mM, pH 7.4)	10	3000× *g*/10 min	[103]
Tris-HCl (10 mM, pH 7.4)	10	4000× *g*/10 min	[104]
Tris-HCl (100 mM, pH 7.4)	10	1400× *g*/10 min	[98]
KCl 1.15% (*w/v*) with Triton X-100 0.1% (*w/v*)	10	5000× *g*/25 min	[105]
Phosphate buffer (50 mM, pH 7.4)	10	10,000× *g*/20 min	[106]
Tris-HCl (10 mM, pH 7.4)	20	3600× *g*/10 min	[102]
Saline solution	10	3000× *g*/10 min	[107]
Phosphate buffer (10 mM, pH 7.4)	14	10,000× *g*/10 min	[108]
Rat liver mitochondria			
Tris-KCl (-)	10	2160 rpm/15 min—discard pellet 8600 rpm /15 min—pellet used	[97]
HEPES buffer (10 mM, pH 7.4) w/ Sucrose 25 mM and Protease Inhibitor Cocktail, 0.5% (*v/v*)	ns	1000× *g*/30 min—discard pellet 10,000× *g*/20 min—pellet used	[109]
Rat liver microsomes			
Tris-malate buffer (5 mM, pH 7.4) with KCl 1.15% (*w/v*)	ns	15,000× *g*/15 min—discard pellet 100,000× *g*/1 h—pellet used	[93,95]
Tris-HCl (50 mM, pH 7.4) with KCl 150 mM	ns	10,000× *g*/20 min—discard pellet 100,000× *g*/1 h—pellet used	[96]
HEPES buffer (10 mM, pH 7.4) w/Sucrose, 25 mM and Protease Inhibitor Cocktail, 0.5% (*v/v*)	ns	1000× *g*/30 min—discard pellet 10,000× *g*/20 min—discard pellet 100,000× *g*/30 min—pellet used	[109]

**Table 2:** Overview of assay conditions used in models with rat-derivatives as substrate. Unless otherwise stated, TBARS were measured at the mentioned endpoints. NP—natural product; ns—not stated; AscH_2_—ascorbic acid; SNP—sodium nitroprusside; AscH^–^—ascorbate anion; G6P—glucose-6-phosphate; G6Pdhase—G6P dehydrogenase; ADP: adenosine diphosphate; t-BOOH: tert-butylhydroperoxide; NADPH: reduced nicotinamide adenine dinucleotide phosphate; DMSO: dimethyl sulfoxide; TBARS: thiobarbituric acid Reactive Species.

Substrate		Oxidative Trigger	Concentration	NP Inclusion	Incubation-Detection System	Ref.
Type	Concentration	Type				
Rat liver homogenate	5% (*w/v*)	Fe^2+^/AscH_2_	0.16 mM/0.06 mM	ns	TBARS (1 h at 37 °C)	[94]
	3.3% (*w/v*)	Fe^3+^/ADP/AscH_2_	0.1 mM/1.7 mM/0.5 mM	ns	TBARS (1 h at 37 °C)	[94]
	10 mg protein/mL	none (O_2_)	ns	Dissolved in water or DMSO	TBARS (15 min at 37 °C)	[83,84,85,86]
	0.5 mg protein/mL	t-BOOH	3 mM	Dissolved in water or DMSO	Chemiluminescence measured online over time, at 30 °C	[83,84,85,86]
	3.3% (*w/v*)	Fe^2+^/SNP	ns	ns	TBARS (1 h at 37 °C)	[98]
	2.5% (*w/v*)	Fe^3+^	10 µM	5% (*v*/*v*), dissolved in DMSO	TBARS (30 min at 37 °C)	[100]
	ns	Fe^3+^/citrate/AscH^−^	ns	ns	TBARS (ns)	[101]
	ns	Fe^2+^	10 µM	ns	TBARS (1 h at 37 °C)	[102]
Rat liver mitochondria	1 mg protein/mL	Fe^2+^/AscH_2_	10 mM/200 mM	ns	TBARS (20 min at 37 °C)	[97]
	0.87% (*w/v*)	Fe^2+^/AscH_2_	1.3 mM/4.3 µM	ns	TBARS (1 h at 37 °C)	[109]
Rat liver microsomes	0.2 mg protein/mL	Fe^2+^/AscH_2_/ADP	1.2 µM/5 µM/0.4 mM	0.1% (*v*/*v*) dissolved in ethanol	HPLC-TBARS along time, 30 °C	[110]
	0.2 mg protein/mL	Fe^2+^/NADPH	5 nM/60 µM	4% (*v*/*v*) dissolved in water	TBARS (30 min at 37 °C)	[95]
	0.25 mg protein/mL	(post heat-inactivation) Fe^3+^/AscH^−^/ADP	1 µM/50 µM/40 µM	ns	TBARS (15 min at 37 °C)	[93]
	0.25 mg protein/mL	NADP/G6P/G6Pdhase	0.2 mM/3 mM/1 U	ns	TBARS (15 min at 37 °C)	[93]
	0.25 mg protein/mL	Fe^2+^/AscH^–^	2.5 µM/0.5 mM	5% (*v*/*v*) dissolved in DMSO	TBARS (30 min at 37 °C)	[96]
Rat brain homogenate	ns	Fe^2+^	10 µM	ns	TBARS (1 h at 37 °C)	[102]
	3.3% (*w/v*)	Fe^2+^/SNP	ns	ns	TBARS (1 h at 37 °C)	[98]
	15% (*w/v*)	Fe^2+^	0.4 mM	ns	TBARS (45 min at 37 °C)	[108]
	ns	SNP	7 mM	ns	TBARS (1 h at 37 °C)	[107]
	0.9% (*w/v*)	Fe^3+^/H_2_O_2_	0.3 mM/60 µM	8.6% (*v/v*) dissolved in acetone	TBARS (1 h at 37 °C)	[105]
	3.1% (*w/v)*	Fe^3+^/H_2_O_2_	12.5 µM/3.1 µM	ns	TBARS (30 min at 37 °C)	[106]
	10% (*w/v*)	SNP	5 µM	ns	TBARS (1 h at 37 °C)	[104]
	10% (*w/v*)	Fe^2+^	10 µM	ns	TBARS (1 h at 37 °C)	[104]
	10% (*w/v*)	Quinolinic acid	1 mM	ns	TBARS (1 h at 37 °C)	[103]
	10% (*w/v*)	SNP	5 µM	ns	TBARS (1 h at 37 °C)	[103]
	10% (*w/v*)	none (O_2_)	ns	50% (*v*/*v*) dissolved in water	TBARS (40 min at 37 °C)	[111]

**Table 4:** Approaches to the dispersion of linoleic acid in an aqueous media. LA—linoleic acid; PB—phosphate buffer; NP—natural product.

Main Solvent in Reactional Medium	Method of Dispersion	Ref.
Water	Emulsification of LA in PB 20 mM pH 7, using Tween-20 (5.6 µg/mL LA and 5.6 µg/mL Tween-20) Mixture of emulsion in PB, water and sample dissolved in 80% methanol	[70]
	Dissolution of LA (8 mM) in Borate buffer 50 mM pH 9 Mixture of previous solution with FeSO_4_, EDTA, H_2_O_2_, sample dissolved in methanol and PB 0.4 M pH 6.75	[126]
	Dissolution of sodium linoleate in water (16 mM) Mixture of previous solution in PB 50 mM pH 7.4	[127]
	Dissolution of LA in ethanol, 2.5% (*v/v*) Mixture of previous solution with distilled water, PB 50 mM pH 7 and sample	[74]
Ethanol	Dissolution of LA (1.3% (*v/v*)) and NP in ethanol:water 3:1 (*v/v*)	[76]

## References

[1] Falade A.O., Oboh G. (2015). Thermal oxidation induces lipid peroxidation and changes in the physicochemical properties and β-carotene content of arachis oil. Int. J. Food Sci..

[2] Brothersen C., McMahon D.J., Legako J., Martini S. (2016). Comparison of milk oxidation by exposure to LED and fluorescent light. J. Dairy Sci..

[3] Breheny D. (2012). Environmental reactive oxygen species and cancer. Systems Biology of Free Radicals and Antioxidants.

[4] Murphy M.P. (2009). How mitochondria produce reactive oxygen species. Biochem. J..

[5] Agmon E., Stockwell B.R. (2017). Lipid homeostasis and regulated cell death. Curr. Opin. Chem. Biol..

[6] Gaschler M.M., Stockwell B.R. (2017). Lipid peroxidation in cell death. Biochem. Biophys. Res. Commun..

[7] Suresh D.R., Annam V. (2013). Lipid peroxidation and total antioxidant capacity in health and disease—Pathophysiology and markers: An overview. Int. J. Med. Sci. Public Heal..

[8] Halliwell B. (2000). Lipid peroxidation, antioxidants and cardiovascular disease: How should we move forward?. Cardiovasc. Res..

[9] Bowe W.P., Logan A.C. (2010). Clinical implications of lipid peroxidation in acne vulgaris: Old wine in new bottles. Lipids Health Dis..

[10] Ramana K.V., Srivastava S., Singhal S.S. (2017). Lipid Peroxidation Products in Human Health and Disease 2016. Oxid. Med. Cell. Longev..

[11] Barrera G. (2012). Oxidative Stress and Lipid Peroxidation Products in Cancer Progression and Therapy. ISRN Oncol..

[12] Celi P. (2010). The role of oxidative stress in small ruminants’ health and production. Rev. Bras. Zootec..

[13] Nam T.G. (2011). Lipid peroxidation and its toxicological implications. Toxicol. Res..

[14] Ahmed M., Pickova J., Ahmad T., Liaquat M., Farid A., Jahangir M. (2016). Oxidation of Lipids in Foods. Sarhad J. Agric..

[15] Velasco J., Dobarganes C., Márquez-Ruiz G. (2010). Oxidative rancidity in foods and food quality. Chemical Deterioration and Physical Instability of Food and Beverages.

[16] Ory R.L., St. Angelo A.J., Gwo Y.Y., Flick G.J., Mod R.R. (2013). Oxidation-Induced Changes in Foods. Chemical Changes in Food During Processing.

[17] Chen C., Pearson A.M., Gray J.I. (1992). Effects of synthetic antioxidants (BHA, BHT and PG) on the mutagenicity of IQ-like compounds. Food Chem..

[18] Lorenzo J.M., Munekata P.E.S., Baldin J.C., Franco D., Domínguez R., Trindade M.A. (2017). The Use of Natural Antioxidants to Replace Chemical Antioxidants in Foods. Strategies for Obtaining Healthier Foods.

[19] Nahas R.I. (2012). Natural antioxidants as food and beverage ingredients. Natural Food Additives, Ingredients and Flavourings.

[20] Bonilla J., Atares L., Chiralt A., Vargas M. (2012). Recent Patents on the Use of Antioxidant Agents in Food. Recent Pat. Food Nutr. Agric..

[21] Kumar Y., Yadav D.N., Ahmad T., Narsaiah K. (2015). Recent Trends in the Use of Natural Antioxidants for Meat and Meat Products. Compr. Rev. Food Sci. Food Saf..

[22] Pokorný J., Yanishlieva N., Gordon M. (2001). Antioxidants in food: Practical applications.

[23] Karakaya M., Bayrak E., Ulusoy K. (2011). Use of Natural Antioxidants in Meat and Meat Products. J. Food Sci. Eng..

[24] Oswell N.J., Thippareddi H., Pegg R.B. (2018). Practical use of natural antioxidants in meat products in the U.S.: A review. Meat Sci..

[25] Lü J.M., Lin P.H., Yao Q., Chen C. (2010). Chemical and molecular mechanisms of antioxidants: Experimental approaches and model systems. J. Cell. Mol. Med..

[26] Alam M.N., Bristi N.J., Rafiquzzaman M. (2013). Review on in vivo and in vitro methods evaluation of antioxidant activity. Saudi Pharm. J..

[27] Apak R., Özyürek M., Güçlü K., Çapanoğlu E. (2016). Antioxidant Activity/Capacity Measurement. 1. Classification, Physicochemical Principles, Mechanisms, and Electron Transfer (ET)-Based Assays. J. Agric. Food Chem..

[28] Apak R., Güçlü K., Demirata B., Özyürek M., Çelik S., Bektaşoğlu B., Berker K., Özyurt D. (2007). Comparative Evaluation of Various Total Antioxidant Capacity Assays Applied to Phenolic Compounds with the CUPRAC Assay. Molecules.

[29] Moon J.K., Shinamoto T. (2009). Antioxidant assays for plant and food components. J. Agric. Food Chem..

[30] Yin H., Xu L., Porter N.A. (2011). Free Radical Lipid Peroxidation: Mechanisms and Analysis. Chem. Rev..

[31] Johnson D.R., Decker E.A. (2015). The Role of Oxygen in Lipid Oxidation Reactions: A Review. Annu. Rev. Food Sci. Technol..

[32] Ayala A., Muñoz M.F., Argüelles S. (2014). Lipid peroxidation: Production, metabolism, and signaling mechanisms of malondialdehyde and 4-hydroxy-2-nonenal. Oxid. Med. Cell. Longev..

[33] Kanner J., German J.B., Kinsella J.E. (1987). Initiation of Lipid Peroxidation in Biological Systems. Crit. Rev. Food Sci. Nutr..

[34] Pryor W.A. (2003). Lipid peroxidation in biological systems. Free Radic. Biol. Med..

[35] Girotti A.W. (1998). Lipid hydroperoxide generation, turnover, and effector action in biological systems. J. Lipid Res..

[36] Dix T.A., Aikens J. (1993). Mechanisms and Biological Relevance of Lipid Peroxidation Initiation. Chem. Res. Toxicol..

[37] Gutteridge J.M.C., Halliwell B. (1990). The measurement and mechanism of lipid peroxidation in biological systems. Trends Biochem. Sci..

[38] Kerr B.J., Kellner T.A., Shurson G.C. (2015). Characteristics of lipids and their feeding value in swine diets. J. Anim. Sci. Biotechnol..

[39] Grune T. (2005). Free Radicals and Diseases: Gene Expression, Cellular Metabolism and Pathophysiology.

[40] Haber F., Weiss J. (1934). The catalytic compensation of hydrogen peroxide by iron salts. Proc. R. Soc. London. Ser. A Math. Phys. Sci..

[41] Choe E., Min D.B. (2006). Chemistry and reactions of reactive oxygen species in foods. Crit. Rev. Food Sci. Nutr..

[42] Bolisetty S., Jaimes E.A. (2013). Mitochondria and reactive oxygen species: Physiology and pathophysiology. Int. J. Mol. Sci..

[43] Galatro A., González P.M., Malanga G., Robello E., Piloni N.E., Puntarulo S. (2013). Nitric oxide and membrane lipid peroxidation in photosynthetic and non-photosynthetic organisms under several stress conditions. Front. Physiol..

[44] Gisone P., Dubner D., Del Rosario Pérez M., Michelin S., Puntarulo S. (2004). The role of nitric oxide in the radiation-induced effects in the developing brain. In Vivo.

[45] Hunt J.P., Taube H. (1952). The Photochemical Decomposition of Hydrogen Peroxide. Quantum Yields, Tracer and Fractionation Effects. J. Am. Chem. Soc..

[46] Carlsen C.U., Møller J.K.S., Skibsted L.H. (2005). Heme-iron in lipid oxidation. Coord. Chem. Rev..

[47] Choe E., Min D.B. (2006). Mechanisms and factors for edible oil oxidation. Compr. Rev. Food Sci. Food Saf..

[48] Crean C., Shao J., Yun B.H., Geacintov N.E., Shafirovich V. (2009). The role of one-electron reduction of lipid hydroperoxides in causing DNA damage. Chemistry.

[49] Zanardi E., Battaglia A., Ghidini S., Conter M., Badiani A., Ianieri A. (2009). Lipid oxidation of irradiated pork products. LWT Food Sci. Technol..

[50] Reichart G., Mayer J., Zehm C., Kirschstein T., Tokay T., Lange F., Baltrusch S., Tiedge M., Fuellen G., Ibrahim S. (2019). Mitochondrial complex IV mutation increases reactive oxygen species production and reduces lifespan in aged mice. Acta Physiol..

[51] Fenton H.J.H. (1984). Oxidation of tartaric acid in presence of iron. J. Chem. Soc..

[52] Min D.B., Boff J.M. (2002). Chemistry and Reaction of Singlet Oxygen in Foods. Compr. Rev. Food Sci. Food Saf..

[53] Haase G., Dunkley W.L. (1969). Ascorbic acid and copper in linoleate oxidation. II. Ascorbic acid and copper as oxidation catalysts. J. Lipid Res..

[54] Ivanova I.P., Trofimova S.V., Piskarev I.M. (2014). Evaluation of prooxidant properties of ascorbic acid. Biophysics.

[55] Chepda T., Perier C., Chamson A., Frey J. (1999). Effets pro- et antioxydants de l’ascorbate. Nutr. Clin. Métabolisme.

[56] Mozuraityte R., Rustad T., Storrø I. (2008). The role of iron in peroxidation of polyunsaturated fatty acids in liposomes. J. Agric. Food Chem..

[57] De Groot H., Noll T. (1986). The crucial role of low steady state oxygen partial pressures in haloalkane free-radical-mediated lipid peroxidation. Possible implications in haloalkane liver injury. Biochem. Pharmacol..

[58] Ingold K.U., Howard J.A. (1969). Absolute rate constants for hydrocarbon autoxidation. XVII. The oxidation of some cyclic ethers. Can. J. Chem..

[59] Devasagayam T.P.A., Boloor K.K., Ramasarma T. (2003). Methods for estimating lipid peroxidation: An analysis of merits and demerits. Indian J. Biochem. Biophys..

[60] Vossen R.C.R.M., van Dam-Mieras M.C.E., Hornstra G., Zwaal R.F.A. (1993). Continuous monitoring of lipid peroxidation by measuring conjugated diene formation in an aqueous liposome suspension. Lipids.

[61] Riely C.A., Cohen G., Lieberman M. (1974). Ethane evolution: A new index of lipid peroxidation. Science.

[62] Lemoyne M., Van Gossum A., Kurian R., Ostro M., Axler J., Jeejeebhoy K.N. (1987). Breath pentane analysis as an index of lipid peroxidation: A functional test of vitamin E status. Am. J. Clin. Nutr..

[63] Ghani M.A., Barril C., Bedgood D.R., Prenzler P.D. (2017). Measurement of antioxidant activity with the thiobarbituric acid reactive substances assay. Food Chem..

[64] Grotto D., Maria L.S., Valentini J., Paniz C., Schmitt G., Garcia S.C., Pomblum V.J., Rocha J.B.T., Farina M. (2009). Importance of the lipid peroxidation biomarkers and methodological aspects FOR malondialdehyde quantification. Quim. Nova.

[65] Jardine D., Antolovich M., Prenzler P.D., Robards K. (2002). Liquid chromatography-mass spectrometry (LC-MS) investigation of the thiobarbituric acid reactive substances (TBARS) reaction. J. Agric. Food Chem..

[66] Du Z., Bramlage W.J. (1992). Modified Thiobarbituric Acid Assay for Measuring Lipid Oxidation in Sugar-Rich Plant Tissue Extracts. J. Agric. Food Chem..

[67] El-Saadani M., Esterbauer H., El-Sayed M., Goher M., Nassar A.Y., Jürgens G. (1989). A spectrophotometric assay for lipid peroxides in serum lipoproteins using a commercially available reagent. J. Lipid Res..

[68] Lips A., Chapman R.A., McFarlane W.D. (1943). The application of the ferric thiocyanate method to the determination of incipient rancidity in fats and oils. Oil Soap.

[69] Yen G.C., Hsieh C.L. (1998). Antioxidant Activity of Extracts from Du-zhong (Eucommia ulmoides) toward Various Lipid Peroxidation Models in Vitro. J. Agric. Food Chem..

[70] Jakovljević V.D., Vrvić M.M., Vrbničanin S., Sarić-Krsmanović M. (2018). Phytochemical, Free Radical Scavenging and Antifungal Profile of Cuscuta campestris Yunck. Seeds. Chem. Biodivers..

[71] Nabavi S.F., Nabavi S.M., Ebrahimzadeh M.A. (2016). Antioxidant activity of hydro-alcoholic extracts of 4 citrus species flower. Prog. Nutr..

[72] Assadpour S., Nabavi S.M., Nabavi S.F., Dehpour A.A., Ebrahimzadeh M.A. (2016). In vitro antioxidant and antihemolytic effects of the essential oil and methanolic extract of Allium rotundum L.. Eur. Rev. Med. Pharmacol. Sci..

[73] China R., Dutta S., Sen S., Chakrabarti R., Bhowmik D., Ghosh S., Dhar P. (2011). In vitro Antioxidant Activity of Different Cultivars of Banana Flower (Musa paradicicus L.) Extracts Available in India. J. Food Sci..

[74] Liu C., Shan Y., Yin X., Li Q. (2013). Antioxidative Capacity of Proanthocyanidins from China Bitter Humulus lupulus in Vitro. J. Am. Soc. Brew. Chem..

[75] Li X.R., Chi C.F., Li L., Wang B. (2017). Purification and identification of antioxidant peptides from protein hydrolysate of scalloped hammerhead (Sphyrna lewini) cartilage. Mar. Drugs.

[76] Freitas C.S., Da Silva G.A., Perrone D., Vericimo M.A., Dos S., Baião D., Pereira P.R., Paschoalin V.M.F., Del Aguila E.M. (2019). Recovery of antimicrobials and bioaccessible isoflavones and phenolics from soybean (glycine max) meal by aqueous extraction. Molecules.

[77] Ozen T., Yenigun S., Altun M., Demirtas I. (2017). Phytochemical Constituents, ChEs and Urease Inhibitions, Antiproliferative and Antioxidant Properties of Elaeagnus umbellata Thunb. Comb. Chem. High Throughput Screen..

[78] Saura-Calixto F. (1998). Antioxidant Dietary Fiber Product: A New Concept and a Potential Food Ingredient. J. Agric. Food Chem..

[79] Repetto M., Semprine J., Boveris A. (2012). Lipid Peroxidation: Chemical Mechanism, Biological Implications and Analytical Determination. Lipid Peroxidation.

[80] Kohno Y., Sakamoto O., Tomita K., Horii I., Miyazawa T. (1991). Determination of Human Skin Surface Lipid Peroxides by Chemiluminescence-HPLC. J. Jpn. Oil Chem. Soc..

[81] Zhang J.R., Cazers A.R., Lutzke B.S., Hall E.D. (1995). HPLC-chemiluminescence and thermospray LC/MS study of hydroperoxides generated from phosphatidylcholine. Free Radic. Biol. Med..

[82] Miyazawa T. (1989). Determination of phospholipid hydroperoxides in human blood plasma by a chemiluminescence-HPLC assay. Free Radic. Biol. Med..

[83] Desmarchelier C., Mongelli E., Coussio J., Ciccia G. (1997). Evaluation of the in vitro antioxidant activity in extracts of Uncaria tomentosa (Willd.) DC. Phyther. Res..

[84] Mongelli E., Desmarchelier C., Talou J.R., Coussio J., Ciccia G. (1997). In vitro antioxidant and cytotoxic activity of extracts of Baccharis coridifolia DC. J. Ethnopharmacol..

[85] Desmarchelier C., Coussio J., Ciccia G. (1998). Antioxidant and free radical scavenging effects in extracts of the medicinal herb Achyrocline satureioides (Lam.) DC. (“marcela”). Braz. J. Med. Biol. Res..

[86] Desmarchelier C., Lisboa Romão R., Coussio J., Ciccia G. (1999). Antioxidant and free radical scavenging activities in extracts from medicinal trees used in the “Caatinga” region in northeastern Brazil. J. Ethnopharmacol..

[87] Wills E.D. (1969). Lipid peroxide formation in microsomes. General considerations. Biochem. J..

[88] Wills E.D., Bartholomew S. (1966). Mechanisms of Lipid Peroxide Formation in Animal Tissues. Biochem. J.

[89] Kornbrust D.J., Mavis R.D. (1980). Microsomal Lipid Peroxidation. Mol. Pharmacol..

[90] Kornbrust D.J., Mavis R.D. (1980). Relative susceptibility of microsomes from lung, heart, liver, kidney, brain and testes to lipid peroxidation: Correlation with vitamin E content. Lipids.

[91] Zhao Y., Wang X., Kawai M., Liu J., Liu M., Mori A. (1995). Antioxidant activity of Chinese ant extract preparations. Acta Med. Okayama.

[92] De Las Heras B., Slowing K., Benedí J., Carretero E., Ortega T., Toledo C., Bermejo P., Iglesias I., Abad M.J., Gómez-Serranillos P. (1998). Antiinflammatory and antioxidant activity of plants used in traditional medicine in Ecuador. J. Ethnopharmacol..

[93] Hanna A.N., Sharma H.M., Kauffman E.M., Newman H.A.I. (1994). In vitro and in vivo inhibition of microsomal lipid peroxidation by MA-631. Pharmacol. Biochem. Behav..

[94] Jose J.K., Kuttan R. (1995). Antioxidant Activity of Emblica officinalis. J. Clin. Biochem. Nutr..

[95] Kaizu H., Sasaki M., Nakajima H., Suzuki Y. (1993). Effect of Antioxidative Lactic Acid Bacteria on Rats Fed a Diet Deficient in Vitamin, E. J. Dairy Sci..

[96] Kim S.W., Park S.S., Min T.J., Yu K.H. (1999). Antioxidant activity of ergosterol peroxide (5,8-epidioxy-5α,8α- ergosta-6,22e-dien-3β-ol) in Armillariella mellea. Bull. Korean Chem. Soc..

[97] Zhu M., Chang Q., Wong L.K., Chong F.S., Li R.C. (1999). Triterpene antioxidants from Ganoderma lucidum. Phyther. Res..

[98] Sabir S.M., Singh D., Rocha J.B.T. (2015). In Vitro Antioxidant Activity of S-Carvone Isolated from Zanthoxylum alatum. Pharm. Chem. J..

[99] Gupta S., Adak S., Rajak R.C., Banerjee R. (2016). In vitro efficacy of Bryophyllum pinnatum leaf extracts as potent therapeutics. Prep. Biochem. Biotechnol..

[100] Sharma U.K., Sharma A.K., Pandey A.K. (2016). Medicinal attributes of major phenylpropanoids present in cinnamon. BMC Complement. Altern. Med..

[101] Dalvi L.T., Moreira D.C., Alonso A., de Avellar I.G.J., Hermes-Lima M. (2018). Antioxidant activity and mechanism of commercial Rama Forte persimmon fruits (Diospyros kaki). PeerJ.

[102] da Cunha F.A.B., Waczuk E.P., Duarte A.E., Barros L.M., Elekofehinti O.O., Matias E.F.F., da Costa J.G.M., Sanmi A.A., Boligon A.A., da Rocha J.B.T. (2016). Cytotoxic and antioxidative potentials of ethanolic extract of Eugenia uniflora L. (Myrtaceae) leaves on human blood cells. Biomed. Pharmacother..

[103] Marques N.F., Stefanello S.T., Froeder A.L.F., Busanello A., Boligon A.A., Athayde M.L., Soares F.A.A., Fachinetto R. (2015). Centella asiatica and Its Fractions Reduces Lipid Peroxidation Induced by Quinolinic Acid and Sodium Nitroprusside in Rat Brain Regions. Neurochem. Res..

[104] de Oliveira D.R., Schaffer L.F., Busanello A., Barbosa C.P., Peroza L.R., de Freitas C.M., Krum B.N., Bressan G.N., Boligon A.A., Athayde M.L. (2015). Silymarin has antioxidant potential and changes the activity of Na^+^/K^+^-ATPase and monoamine oxidase in vitro. Ind. Crops Prod..

[105] Kapravelou G., Martínez R., Andrade A.M., López Chaves C., López-Jurado M., Aranda P., Arrebola F., Cañizares F.J., Galisteo M., Porres J.M. (2014). Improvement of the antioxidant and hypolipidaemic effects of cowpea flours (Vigna unguiculata) by fermentation: Results of in vitro and in vivo experiments. J. Sci. Food Agric..

[106] Moniruzzaman M., Asaduzzaman M., Hossain M.S., Sarker J., Rahman S.M.A., Rashid M., Rahman M.M. (2015). In vitro antioxidant and cholinesterase inhibitory activities of methanolic fruit extract of Phyllanthus acidus. BMC Complement. Altern. Med..

[107] Barut E.N., Barut B., Engin S., Yıldırım S., Yaşar A., Türkiş S., Özel A., Sezen F.S. (2017). Antioxidant capacity, anti-acetylcholinesterase activity and inhibitory effect on lipid peroxidation in mice brain homogenate of Achillea millefolium. Turk. J. Biochem..

[108] Fidelis M., Santos J.S., Escher G.B., Vieira do Carmo M., Azevedo L., Cristina da Silva M., Putnik P., Granato D. (2018). In vitro antioxidant and antihypertensive compounds from camu-camu (Myrciaria dubia McVaugh, Myrtaceae) seed coat: A multivariate structure-activity study. Food Chem. Toxicol..

[109] Raman S., Ganeshan A.K.G., Chen C., Jin C., Li S.H., Chen H.J., Gui Z. (2016). In vitro and In vivo antioxidant activity of flavonoid extracted from mulberry fruit (Morus alba L.). Pharmacogn. Mag..

[110] Rubio-Senent F., de Roos B., Duthie G., Fernández-Bolaños J., Rodríguez-Gutiérrez G. (2015). Inhibitory and synergistic effects of natural olive phenols on human platelet aggregation and lipid peroxidation of microsomes from vitamin E-deficient rats. Eur. J. Nutr..

[111] Gutierrez D.D., Ortega W.M., De Oliveira E., Silva A.M., Muñoz C.Z., Mancini-Filho J., Novoa A.V. (2015). Comparación de las propiedades antioxidantes y contenido de polifenoles de extractos acuosos de las algas marinas Bryothamnion triquetrum y Halimeda opuntia. Ars Pharm..

[112] Gao S., Liu J. (2017). Association between circulating oxidized low-density lipoprotein and atherosclerotic cardiovascular disease. Chronic Dis. Transl. Med..

[113] Khoo H.E., Azlan A., Ismail A., Abas F., Hamid M. (2014). Inhibition of oxidative stress and lipid peroxidation by anthocyanins from defatted Canarium odontophyllum pericarp and peel using in vitro bioassays. PLoS ONE.

[114] Hseu Y.C., Lee C.C., Chen Y.C., Senthil Kumar K.J., Chen C.S., Tsai C.T., Huang H.C., Wang H.M., Yang H.L. (2014). Antrodia salmonea in submerged culture exhibits antioxidant activities in vitro and protects human erythrocytes and low-density lipoproteins from oxidative modification. Food Chem. Toxicol..

[115] Yang L., Kirikoshi J., Sekimoto S., Takasugi M., Fukunaga K., Hosomi R., Hishida A., Kawahara N., Yamagishi T., Arai H. (2015). Effect of Bean Extract of Yabumame (*Amphicarpaea bracteata* (L.) Fernald subsp. *edgeworthii* (Benth.) H.Ohashi) on Low-Density Lipoprotein Oxidation In Vitro. Food Sci. Technol. Res..

[116] Rosenblat M., Volkova N., Borochov-Neori H., Judeinstein S., Aviram M. (2015). Anti-atherogenic properties of Date vs. Pomegranate polyphenols, in-vitro and in-vivo, in atherosclerotic mice: Beneficial role for the combination. Food Funct..

[117] Sharma H.M., Hanna A.N., Kauffman E.M., Newman H.A.I. (1992). Inhibition of human low-density lipoprotein oxidation in vitro by Maharishi Ayur-Veda herbal mixtures. Pharmacol. Biochem. Behav..

[118] dos Santos M.M., Prestes A.S., de Macedo G.T., Ecker A., Barcelos R.P., Boligon A.A., Souza D., de Bem A.F., da Rocha J.B.T., Barbosa N.V. (2018). Syzygium cumini leaf extract inhibits LDL oxidation, but does not protect the liproprotein from glycation. J. Ethnopharmacol..

[119] Esterbauer H., Striegl G., Puhl H., Rotheneder M. (1989). Continuous monitoring of in vztro oxidation of human low density lipoprotein. Free Radic. Res..

[120] Seng C.K., Abdullah N., Aminudin N. (2017). Antioxidative and Inhibitory Effects of the Fruiting Body of Black Lingzhi Mushroom, Amauroderma rugosum (Agaricomycetes), on LDL Oxidation and HMG-CoA Reductase Activity. Int. J. Med. Mushrooms.

[121] Dairi S., Madani K., Aoun M., Him J.L.K., Bron P., Lauret C., Cristol J.P., Carbonneau M.A. (2014). Antioxidative Properties and Ability of Phenolic Compounds of Myrtus communis Leaves to Counteract In Vitro LDL and Phospholipid Aqueous Dispersion Oxidation. J. Food Sci..

[122] Faisi J., Fattahi A., Raffel N., Hoffmann I., Beckmann M.W., Schrauder M., Dittrich R., Löhberg C. (2018). Effects of pomegranate seed oil and fermented juice polyphenols fraction in different solvents on copper-induced LDL oxidation. CYTA J. Food.

[123] Williams P., Ongsakul M., Proudfoot J., Croft K., Beilin L. (1995). Mangostin Inhibits the Oxidative Modification Of Human Low Density Lipoprotein. Free Radic. Res..

[124] Hartati S., Suparmo, Santoso U., Marsono Y. (2017). Antioxidant activity and in vitro inhibition human plasma LDL oxidation of defatted rice bran var. Menthikwangi extract. Int. Food Res. J..

[125] Gutierrez R.M.P., Baez E. (2014). Evaluation of antidiabetic, antioxidant and antiglycating activities of the Eysenhardtia polystachya. Pharmacogn. Mag..

[126] Brahmi F., Guendouze N., Hauchard D., Okusa P., Kamagaju L., Madani K., Duez P. (2017). Phenolic profile and biological activities of Micromeria graeca (L.) Benth. ex Rchb. Int. J. Food Prop..

[127] Kontogiorgis C., Ntella M., Mpompou L., Karallaki F., Athanasios P., Hadjipavlou-Litina D., Lazari D. (2016). Study of the antioxidant activity of Thymus sibthorpii Bentham (Lamiaceae). J. Enzym. Inhib. Med. Chem..

[128] Kim H.S., Chin K.B. (2017). Evaluation of Antioxidative Activity of Various Levels of Ethanol Extracted Tomato Powder and Application to Pork Patties. Korean J. Food Sci. Anim. Resour..

[129] Banni S., Contini M.S., Angioni E., Deiana M., Dessì M.A., Melis M.P., Carta G., Corongiu F.P. (1996). A novel approach to study linoleic acid autoxidation: Importance of simultaneous detection of the substrate and its derivative oxidation products. Free Radic. Res..

[130] Glória M.B.A., Grulke E.A., Gray J.I. (1993). Effect of type of oxidation on β-carotene loss and volatile products formation in model systems. Food Chem..

[131] Koleva I.I., Van Beek T.A., Linssen J.P.H., Groot A.D., Evstatieva L.N. (2002). Screening of Plant Extracts for Antioxidant Activity: A Comparative Study on Three Testing Methods. Phytochem. Anal..

[132] Roginsky V., Lissi E. (2005). Review of methods to determine chain-breaking antioxidant activity in food. Food Chem..

[133] Laguerre M., Lecomte J., Villeneuve P. (2007). Evaluation of the ability of antioxidants to counteract lipid oxidation: Existing methods, new trends and challenges. Prog. Lipid Res..

[134] Dawidowicz A.L., Olszowy M. (2010). Influence of some experimental variables and matrix components in the determination of antioxidant properties by β-carotene bleaching assay: Experiments with BHT used as standard antioxidant. Eur. Food Res. Technol..

[135] Miller H.E. (1971). A simplified method for the evaluation of antioxidants. J. Am. Oil Chem. Soc..

[136] Marco G.J. (1968). A Rapid Method for Evaluation of Antioxidants. J. Am. Oil Chem. Soc..

[137] Prieto M.A., Rodríguez-Amado I., Vázquez J.A., Murado M.A. (2012). β-Carotene Assay Revisited. Application To Characterize and Quantify Antioxidant and Prooxidant Activities in a Microplate. J. Agric. Food Chem..

[138] Frankel E.N., Finley J.W. (2008). How To Standardize the Multiplicity of Methods To Evaluate Natural Antioxidants. J. Agric. Food Chem..

[139] Ng R.C., Kassim N.K., Yunie Soon Y.Y., Gwendoline Cheng L.E., Yazan S.L., Musa K.H. (2018). Isolation of Carbazole Alkaloids and Coumarins from Aegle marmelos and Murraya koenigii and Their Antioxidant Properties. Sains Malays..

[140] Santos B.C.S., Pires A.S., Yamamoto C.H., Couri M.R.C., Taranto A.G., Alves M.S., Araújo A.L., dos S., de M., de Sousa O.V. (2018). Methyl Chavicol and Its Synthetic Analogue as Possible Antioxidant and Antilipase Agents Based on the In Vitro and In Silico Assays. Oxid. Med. Cell. Longev..

[141] Loizzo M.R., Falco T., Bonesi M., Sicari V., Tundis R., Bruno M. (2018). Ruta chalepensis L. (Rutaceae) leaf extract: Chemical composition, antioxidant and hypoglicaemic activities. Nat. Prod. Res..

[142] Marrelli M., Conforti F., Araniti F., Casacchia T., Statti G. (2018). Seasonal and environmental variability of non-cultivated edible Cichorioideae (Asteraceae). Plant Biosyst. Int. J. Deal. Asp. Plant Biol..

[143] Pant N.C., Joshi K., Kumar M., Singh J.P., Agrawal S. (2018). Evaluation of in vitro antioxidant property and phytochemical contents in different genotypes of fenugreek (Trigonella foenum graecum L.). Ann. Phytomedicine Int. J..

[144] Yilmaz M.A., Ertas A., Yener I., Akdeniz M., Cakir O., Altun M., Demirtas I., Boga M., Temel H. (2018). A comprehensive LC–MS/MS method validation for the quantitative investigation of 37 fingerprint phytochemicals in Achillea species: A detailed examination of A. coarctata and A. monocephala. J. Pharm. Biomed. Anal..

[145] Msaada K., Ben Jemia M., Salem N., Bachrouch O., Sriti J., Tammar S., Bettaieb I., Jabri I., Kefi S., Limam F. (2017). Antioxidant activity of methanolic extracts from three coriander (Coriandrum sativum L.) fruit varieties. Arab. J. Chem..

[146] Chahdoura H., Barreira J.C.M., Adouni K., Mhadhebi L., Calhelha R.C., Snoussi M., Majdoub H., Flamini G., Ferreira I.C.F.R., Achour L. (2017). Bioactivity and chemical characterization of Opuntia macrorhiza Engelm. seed oil: Potential food and pharmaceutical applications. Food Funct..

[147] Elzaawely A.A., Maswada H.F., El-Sayed M.E.A., Ahmed M.E. (2017). Phenolic Compounds and Antioxidant Activity of Rice Straw Extract. Int. Lett. Nat. Sci..

[148] Hui A.C., Foon C.S., Hock C.C. (2017). Antioxidant activities of Elaeis guineensis leaves. J. Oil Palm Res..

[149] Sellimi S., Ksouda G., Benslima A., Nasri R., Rinaudo M., Nasri M., Hajji M. (2017). Enhancing colour and oxidative stabilities of reduced-nitrite turkey meat sausages during refrigerated storage using fucoxanthin purified from the Tunisian seaweed Cystoseira barbata. Food Chem. Toxicol..

[150] Konings A.W.T., Damen J., Trieling W.B. (1979). Protection of liposomal lipids against radiation induced oxidative damage. Int. J. Radiat. Biol..

[151] Aruoma O.I., Spencer J.P.E., Rossi R., Aeschbach R., Khan A., Mahmood N., Munoz A., Murcia A., Butler J., Halliwell B. (1996). An evaluation of the antioxidant and antiviral action of extracts of rosemary and provençal herbs. Food Chem. Toxicol..

[152] Bałasińska B., Troszyńska A. (1998). Total Antioxidative Activity of Evening Primrose (Oenothera paradoxa) Cake Extract Measured in Vitro by Liposome Model and Murine L1210 Cells. J. Agric. Food Chem..

[153] Surendraraj A., Farvin K.H.S., Anandan R. (2013). Antioxidant Potential of Water Hyacinth (Eichornia crassipes): In Vitro Antioxidant Activity and Phenolic Composition. J. Aquat. Food Prod. Technol..

[154] Koduvayur Habeebullah S.F., Nielsen N.S., Jacobsen C. (2010). Antioxidant activity of potato peel extracts in a fish-rapeseed oil mixture and in oil-in-water emulsions. J. Am. Oil Chem. Soc..

[155] Sowmya R., Sachindra N.M. (2012). Evaluation of antioxidant activity of carotenoid extract from shrimp processing byproducts by in vitro assays and in membrane model system. Food Chem..

[156] Akbarzadeh A., Rezaei-Sadabady R., Davaran S., Joo S.W., Zarghami N., Hanifehpour Y., Samiei M., Kouhi M., Nejati-Koshki K. (2013). Liposome: Classification, preparation, and applications. Nanoscale Res. Lett..

[157] Ani V., Naidu K.A. (2011). Antioxidant potential of bitter cumin (Centratherum anthelminticum (L.) Kuntze) seeds in in vitro models. BMC Complement. Altern. Med..

[158] Nieto G., Huvaere K., Skibsted L.H. (2011). Antioxidant activity of rosemary and thyme by-products and synergism with added antioxidant in a liposome system. Eur. Food Res. Technol..

[159] Locatelli M., Travaglia F., Giovannelli L., Coïsson J.D., Bordiga M., Pattarino F., Arlorio M. (2013). Clovamide and phenolics from cocoa beans (Theobroma cacao L.) inhibit lipid peroxidation in liposomal systems. Food Res. Int..

[160] Rosa A., Atzeri A., Deiana M., Scano P., Incani A., Piras C., Cesare Marincola F. (2015). Comparative antioxidant activity and 1H NMR profiling of Mediterranean fruit products. Food Res. Int..

[161] Azofeifa G., Quesada S., Boudard F., Morena M., Cristol J.P., Pérez A.M., Vaillant F., Michel A. (2013). Antioxidant and Anti-inflammatory in Vitro Activities of Phenolic Compounds from Tropical Highland Blackberry (Rubus adenotrichos). J. Agric. Food Chem..

